# Supplementary material for: Expansion of interferon inducible gene pool via USP18 inhibition promotes cancer cell pyroptosis
Source: Nat Commun. 2023 Jan 17;14:251. doi: 10.1038/s41467-022-35348-5 (PMC9842760; doi:10.1038/s41467-022-35348-5)
Supplement: Supplementary file 1 — Supplementary Information [file 41467_2022_35348_MOESM1_ESM.pdf]

Expansion of interferon inducible gene pool via USP18 inhibition promotes cancer cell pyroptosis

Kei-ichiro Arimoto[1], Sayuri Miyauchi[1], Ty D. Troutman[2], Yue Zhang[3], Mengdan Liu[3], Samuel A. Stoner[1], Amanda G. Davis[1], Jun-Bao Fan[1], Yi-Jou Huang[3], Ming Yan[1], Christopher K. Glass[2,4], and Dong-Er Zhang[1,5*]

[1]Moores UCSD Cancer Center, University of California San Diego, La Jolla, USA

[2]Department of Medicine, University of California San Diego, La Jolla, USA

[3] Division of Biological Sciences, University of California San Diego, La Jolla, USA

[4]Department of Cellular and Molecular Medicine, University of California San Diego, La Jolla, USA

[5]Department of Pathology, University of California San Diego, La Jolla, USA

*Correspondence: d7zhang@health.ucsd.edu

Supplementary Information

Supplementary Fig.1
Supplementary Fig.2
Supplementary Fig.3
Supplementary Fig.4
Supplementary Fig.5
Supplementary Fig.6
Supplementary Fig.7
Supplementary Fig.8
Supplementary Fig.9
Supplementary Fig.10
Supplementary Fig.11
Supplementary Fig.12
Supplementary Table 1
Supplementary Table 2
Supplementary Table 3
Supplementary Table 4
Supplementary Table 5
Supplementary Table 6

Supplementary Figure.1 (Arimoto et al)

a

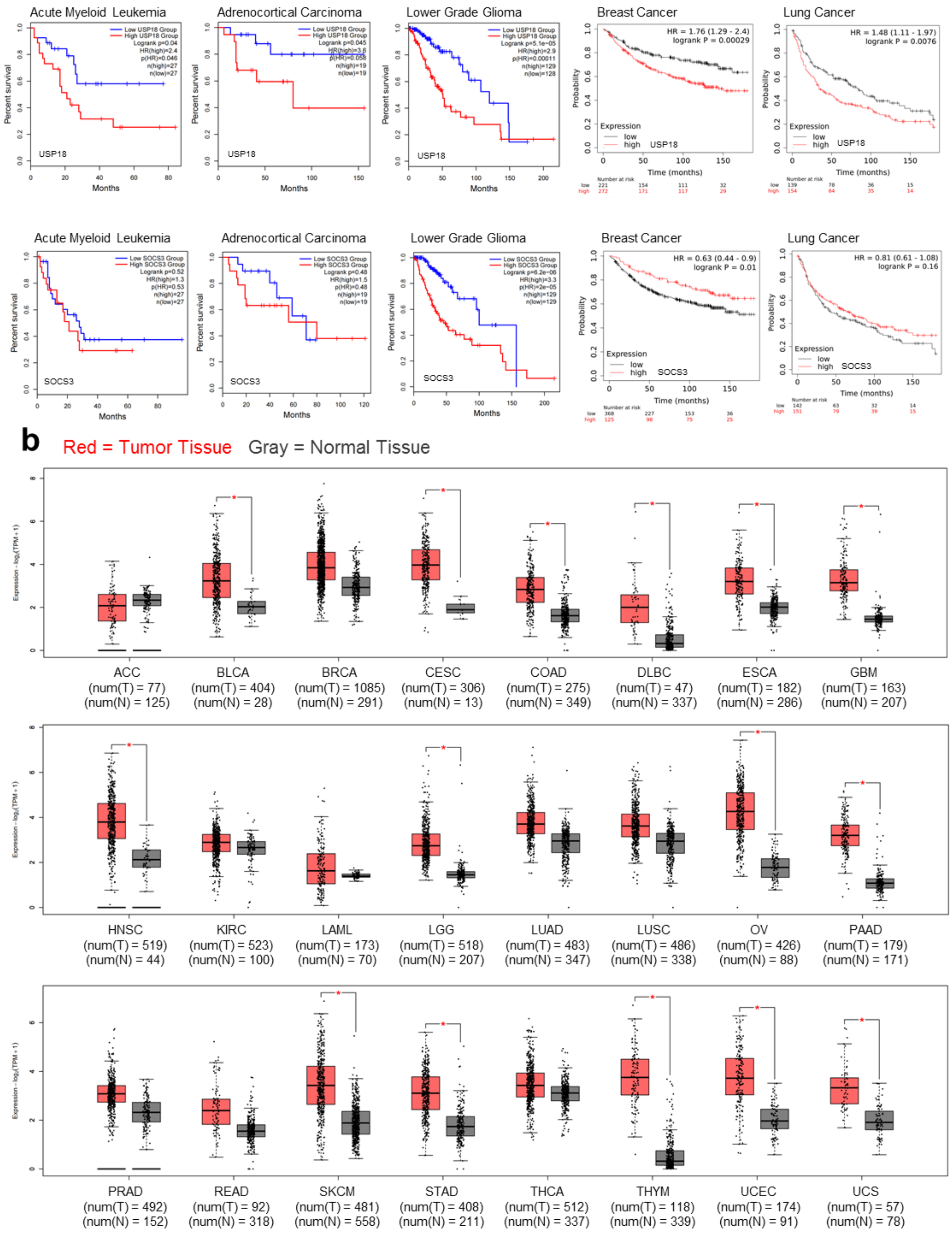

Supplementary Fig. 1.

USP18 expression and associated clinical outcomes

a; Kaplan Meier survival curves for USP18 (*Top*) and SOCS3 (*Bottom*) of the indicated TCGA datasets: KmPlot analysis for Breast Cancer and Lung cancer. HR; Hazard Ratio

b; USP18 expression in numerous cancers compared to normal tissue from GEPIA2 data set including GTEx and TCGA data. Expression (*Y*-axis) is derived from RNA-seq on Log2 scale. Boxplot center represents median, bounds represent 25 and 75%, and whiskers show the minimum or maximum no further than 1.5 times interquartile range from the bound. *p* value was determined by one-way ANOVA. * shows *p*-value < 0.05.

Supplementary Figure.2 (Arimoto et al)

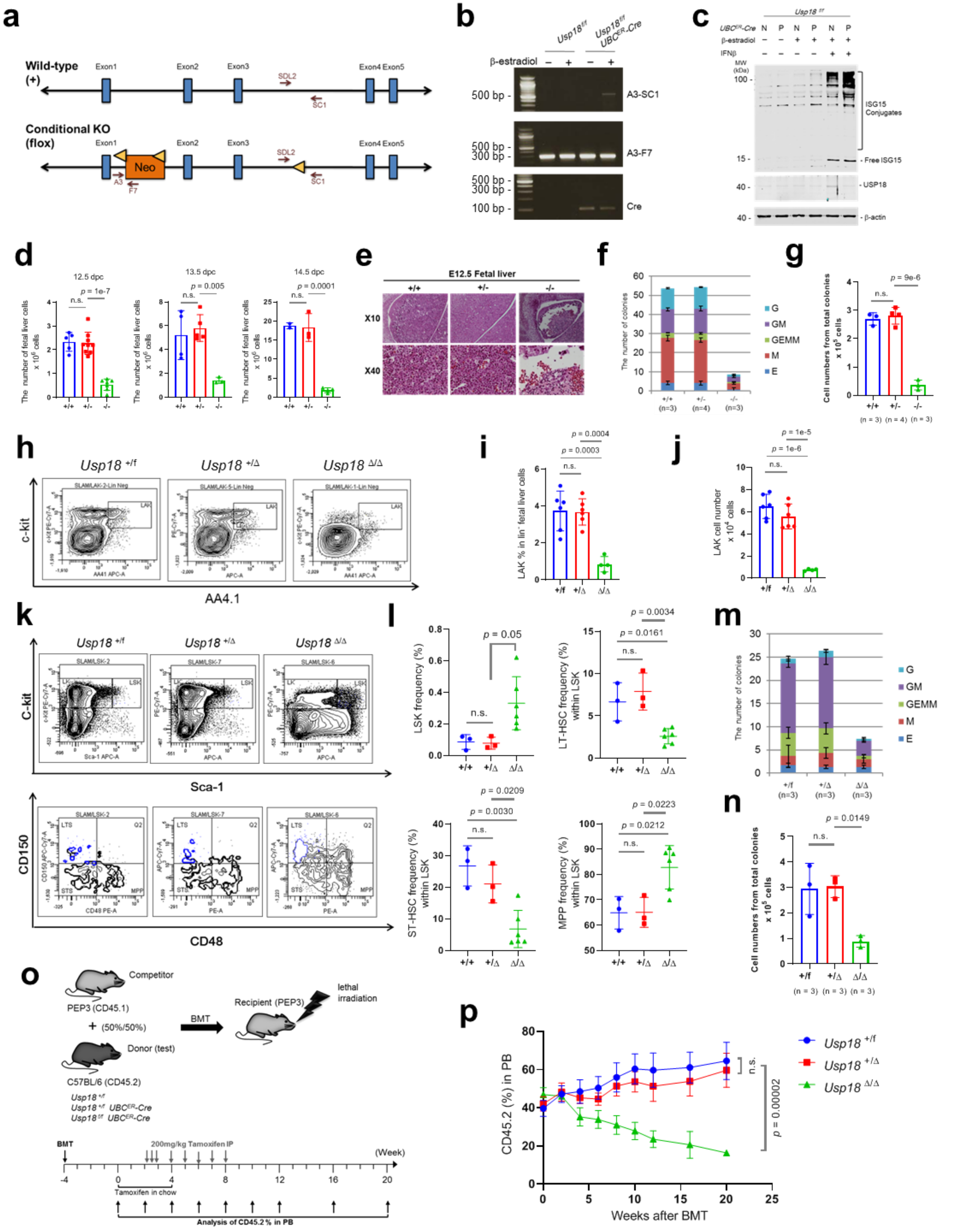

Supplementary Fig. 2.

Two alleles of *Usp18* is required for normal HSC maintenance.

a; Schematic of the strategy for conditional *Usp18* deletion.

b; Verification of the *Usp18* deletion in *Usp18*$^{f/f}$ conditional KO by genotyping PCR.

c; Verification of the USP18 deletion in *Usp18*$^{f/f}$ conditional KO bone marrow cells by western blot.

d; Quantification of fetal liver cells in WT, *Usp18*$^{+/-}$ and *Usp18*$^{-/-}$ mice (E12.5: +/+, +/-, -/-, n = 5, 9, 7, E13.5: n = 4, 5,3, E14.5: n = 2, 3, 4).

e; Representative hematoxylin-Eosin (HE) staining for WT, *Usp18*$^{+/-}$ and *Usp18*$^{-/-}$ fetal liver at E12.5.

f; Colony assay for WT (n =3), *Usp18*$^{+/-}$ (n = 4) and *Usp18*$^{-/-}$ (n = 3) total fetal livers at E12.5.

g; Quantification of total cell number from fetal liver derived colonies of (f).

h; Representative FACS plot of LAK (Lin- AA4.1+ c-Kit +) for *Usp18* $^{+/f}$ (+/f) (n = 6), *Usp18* $^{+/f}$ Vav-iCre (+/Δ) (n = 6), and *Usp18*$^{f/f}$ Vav-iCre (Δ/Δ) (n = 4) fetal liver cells.

i; The frequencies of LAK cells for (h).

j; The absolute number of LAK cells for (h).

k; Representative FACS plot of HSCs from *Usp18* $^{+/f}$, *Usp18* $^{+/\Delta}$ and *Usp18* $^{\Delta/\Delta}$ 8 weeks old mice.

l; The frequencies of LSK, and the frequencies of LT-HSC, ST-HSC and MPP within LSK cells in bone marrow cells from *Usp18* $^{+/f}$ (n = 3), *Usp18* $^{+/\Delta}$ (n = 3) and *Usp18* $^{\Delta/\Delta}$ (n = 6) mice.

m; Colony assay for BM LT-HSC of *Usp18* $^{+/f}$ (n = 3), *Usp18* $^{+/\Delta}$ (n = 3) and *Usp18* $^{\Delta/\Delta}$ (n = 3) mice.

n; Quantification of total cell number from derived colonies of (m).

o; Competitive bone marrow transplantation experimental schematic. Bone marrow cells from PEP3 (CD45.1) competitor and C57BL/6 (CD45.2) background *Usp18* $^{+/f}$ (n = 4), *Usp18* $^{+/f}$ *UBC*^{ER}*-Cre* (n = 4) or *Usp18* $^{f/f}$ *UBC*^{ER}*-Cre* (n = 5) mice was mixed as 50%:50%, followed by transplanted to recipient PEP3 mice.

p; Contribution of CD45.2-expressing (donor) cells to PB in reconstituted mice for (o).

All *p*-values were determined by one-way ANOVA test. All data represent mean ± s.d, except where indicated. n.s. Source data are provided as a Source Data file.

Supplementary Figure.3 (Arimoto et al)

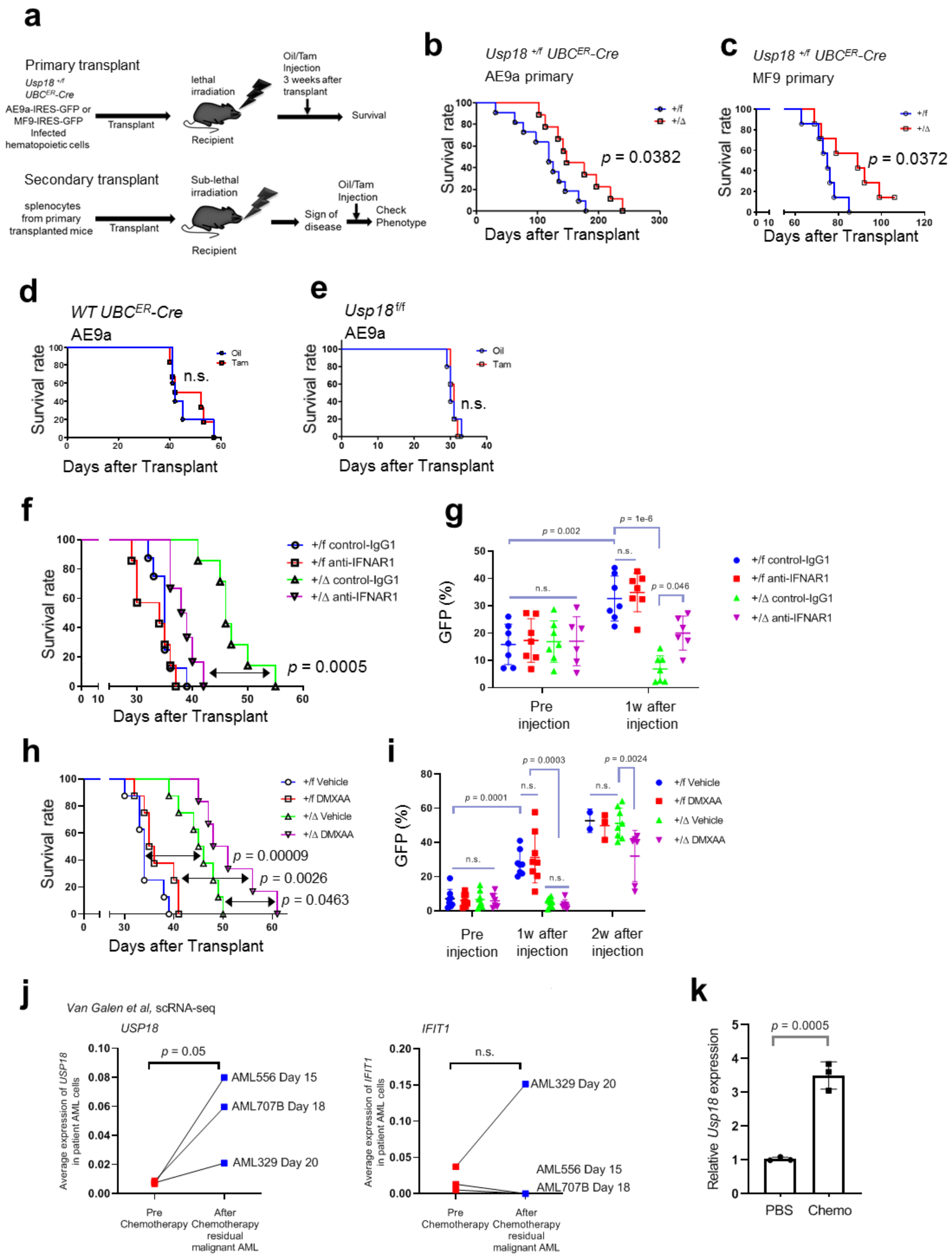

Supplementary Fig. 3.

Inducible *Usp18* deletion delays cancer development in mouse models

a; Schematic strategy for heterozygous deletion of *Usp18* in leukemia primary and secondary transplantation mouse models.

b; *Usp18*$^{+/f}$ *UBC*^{ER}*-Cre* fetal liver cells were infected with MSCV-IRES-GFP AE9a retrovirus and transplanted (i.v.) into lethally irradiated recipient mice. Three weeks after transplantation, oil or tamoxifen was injected (i.p.) daily for 5 days (Oil (+/f) n = 10, Tam (+/Δ) n = 9).

c; Same procedure as (a) using MSCV-IRES-GFP MLL-AF9 retrovirus (+/f n = 7, +/Δ n = 7).

d; Survival of sub-lethally irradiated recipient mice transplanted with WT *UBC*^{ER}*-Cre* AE9a GFP splenocytes from primary bone marrow transplanted mice (n = 5 mice each). After confirming that the mice become sick, oil or tamoxifen was injected 5 times.

e; Same procedure as (d) using *Usp18* $^{f/f}$ AE9a GFP splenocytes (n = 5 mice each).

f; Survival for recipients of *Usp18*$^{+/f \text{ or } +/\Delta}$ *UBC*^{ER}*-Cre* AE9a GFP AMLs with once control IgG1 or anti-IFNAR1 antibody injection (i.v.). (+/f /control IgG1 n = 7, +/f /anti-IFNAR1 n = 7, +/Δ/control IgG1 n = 7, +/Δ/anti-IFNAR1 n = 6).

g; The percentage of GFP+ cells in the PB from (f) were analyzed before and 1 week after oil or tamoxifen injection.

h; Survival for recipients of *Usp18*$^{+/f \text{ or } +/\Delta}$ *UBC*^{ER}*-Cre* AE9a-GFP AMLs with once vehicle or DMXAA injection (i.v.). (+/f/vehicle n = 8, +/f/DMXAA n = 8, +/Δ/vehicle n = 8, +/Δ/DMXAA n = 6).

i; GFP % of (h) in PB were analyzed.

j; scRNA-seq of three AML patients who had been given standard chemotherapy were analyzed. The average expressions of *USP18* and *IFIT1* in AMLs pre- and post-chemotherapy (~ Day 20) are shown.

k; Soon after PBS or chemotherapy were given to AE9a GFP AML bearing mice, GFP+ cells were sorted and the expression of *Usp18* was analyzed by qRT-PCR.

p-value for all survival analysis in Supplementary Fig.3 was determined by log-rank test. One-way ANOVA test for Supplementary Fig.3g, i, two-tailed Student *t*-test for Supplementary Fig.3j-k were used for *p*-values. All data represent mean ± s.d, except where indicated. n.s. = not statistically significant. Source data are provided as a Source Data file.

Supplementary Figure 4 (Arimoto et al)

a

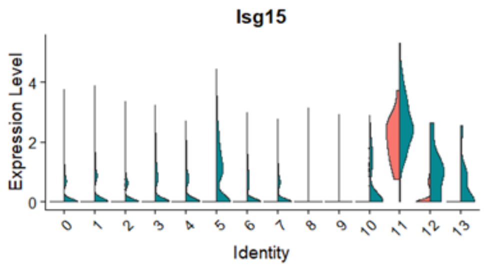

b

Cluster No.	+/f No. of cells	+/f frequency (%)	+/Δ No. of cells	+/Δ frequency (%)	Ratio (+/Δ vs +/f)
0	987	13.84	1101	14.44	1.04
1	881	12.35	1183	15.52	1.26
2	993	13.92	902	11.83	0.85
3	1005	14.09	867	11.37	0.81
4	779	7.04	638	9.72	1.38
5	502	7.04	741	9.72	1.38
6	533	7.47	566	7.42	0.99
7	558	7.82	520	6.82	0.87
8	293	4.11	779	10.22	2.49
9	407	5.71	69	0.91	0.16
10	143	2.01	98	1.29	0.64
11	12	0.17	115	1.51	8.96
12	35	0.49	22	0.29	Excluded, low No. of cells
13	4	0.06	23	0.30	Excluded, low No. of cells

c

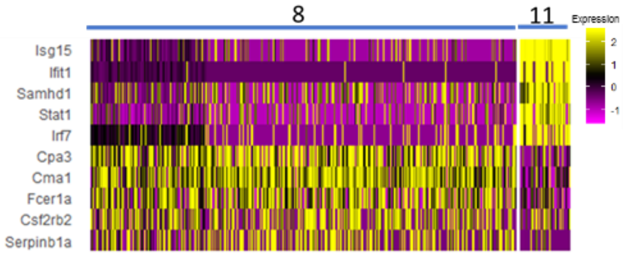

d

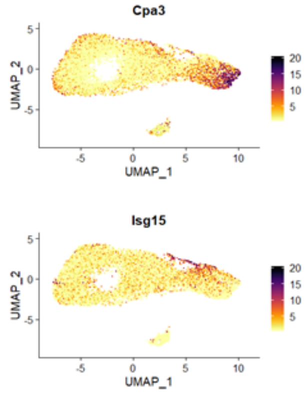

e

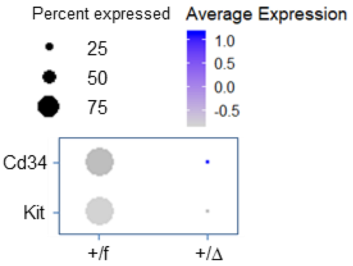

f

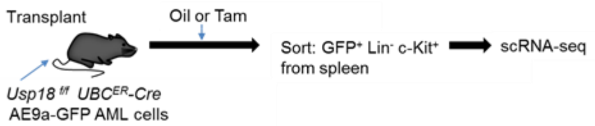

g

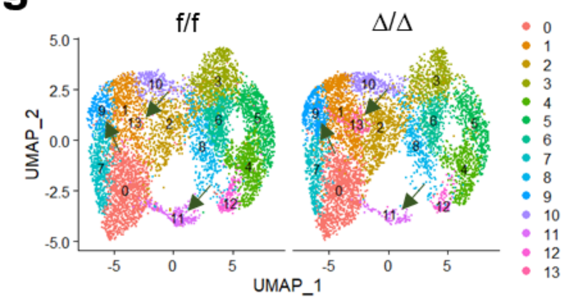

h

Cluster No.	f/f No. of cells	f/f freqiency (%)	Δ/Δ No. of cells	Δ/Δ freqiency (%)	Ratio (Δ/Δ vs f/f)
0	1353	18.01	1210	17.07	0.95
1	638	8.49	931	13.13	1.55
2	646	8.60	846	11.93	1.39
3	720	9.58	547	7.72	0.81
4	710	9.45	530	7.48	0.79
5	741	9.86	497	7.01	0.71
6	609	8.11	437	6.16	0.76
7	539	7.17	467	6.59	0.92
8	438	5.83	392	5.53	0.95
9	291	3.87	469	6.62	1.71
10	287	3.82	363	5.12	1.34
11	307	4.09	112	1.58	0.39
12	215	2.86	109	1.54	0.54
13	19	0.25	179	2.53	9.98

i

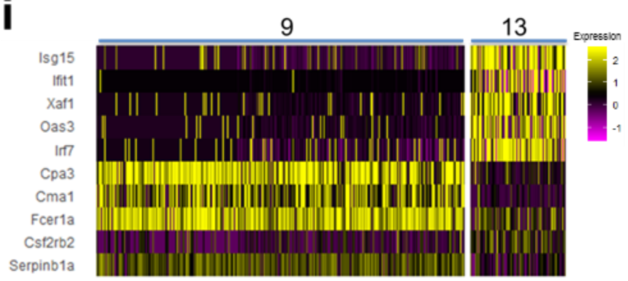

j

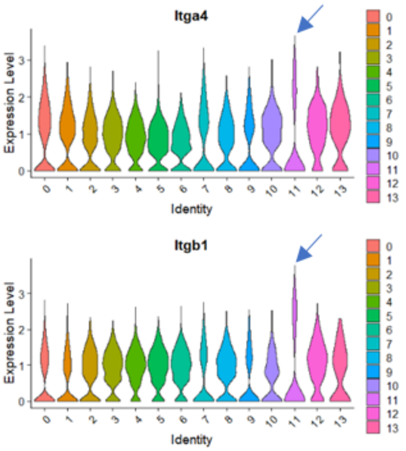

Supplementary Fig. 4.

Single cell RNA-seq analysis of *Usp18*$^{+/f}$ and *Usp18*$^{+/\Delta}$ or *Usp18*$^{f/f}$ and *Usp18*$^{\Delta/\Delta}$ AE9a recipient mice

a; Violin plots of *Isg15* expression in all clusters with or without *Usp18* suppression in scRNA seq analysis of *Usp18*$^{+/f}$ and *Usp18*$^{+/\Delta}$.

b; Table displaying the number of cells in each cluster, frequency among all clusters, and ratio describing changes in cluster frequencies between the two groups of mice (+/f vs +/Δ).

c; Heatmap of phenotypic gene expression in clusters 8 and 11 in scRNA seq analysis of *Usp18*$^{+/f}$ and *Usp18*$^{+/\Delta}$.

d; Feature plots for representative genes in clusters 8 and 11 in scRNA seq analysis of *Usp18*$^{+/f}$ and *Usp18*$^{+/\Delta}$.

e; Dot plot of Cd34 and Kit expression in cluster 9 before and after *Usp18* deletion in scRNA seq analysis of *Usp18*$^{+/f}$ and *Usp18*$^{+/\Delta}$.

f; Single cell RNA-seq of GFP^{+}Lin^{-}c-Kit^{+} cells from *Usp18*$^{f/f}$ and *Usp18*$^{\Delta/\Delta}$ AE9a recipient mice (*Usp18*$^{f/f}$ n = 3, *Usp18*$^{\Delta/\Delta}$ n = 3 mice each pooled).

g; UMAP of sorted GFP^{+}Lin^{-}c-Kit^{+} cells from *Usp18*$^{f/f}$ and *Usp18*$^{\Delta/\Delta}$ AE9a recipient mice. Arrows indicates clusters 9, 11 and 13.

h; Table displaying the number of cells in each cluster, frequency among all clusters, and ratio describing changes in cluster frequencies between the two groups of mice (f/f vs Δ/Δ).

i; Heatmap of phenotypic gene expression in clusters 9 and 13 in scRNA seq analysis of *Usp18*$^{f/f}$ and *Usp18*$^{\Delta/\Delta}$.

j; Violin plots for *Itga4* and *Itgb1* in all clusters.

Supplementary Figure 5 (Arimoto et al)

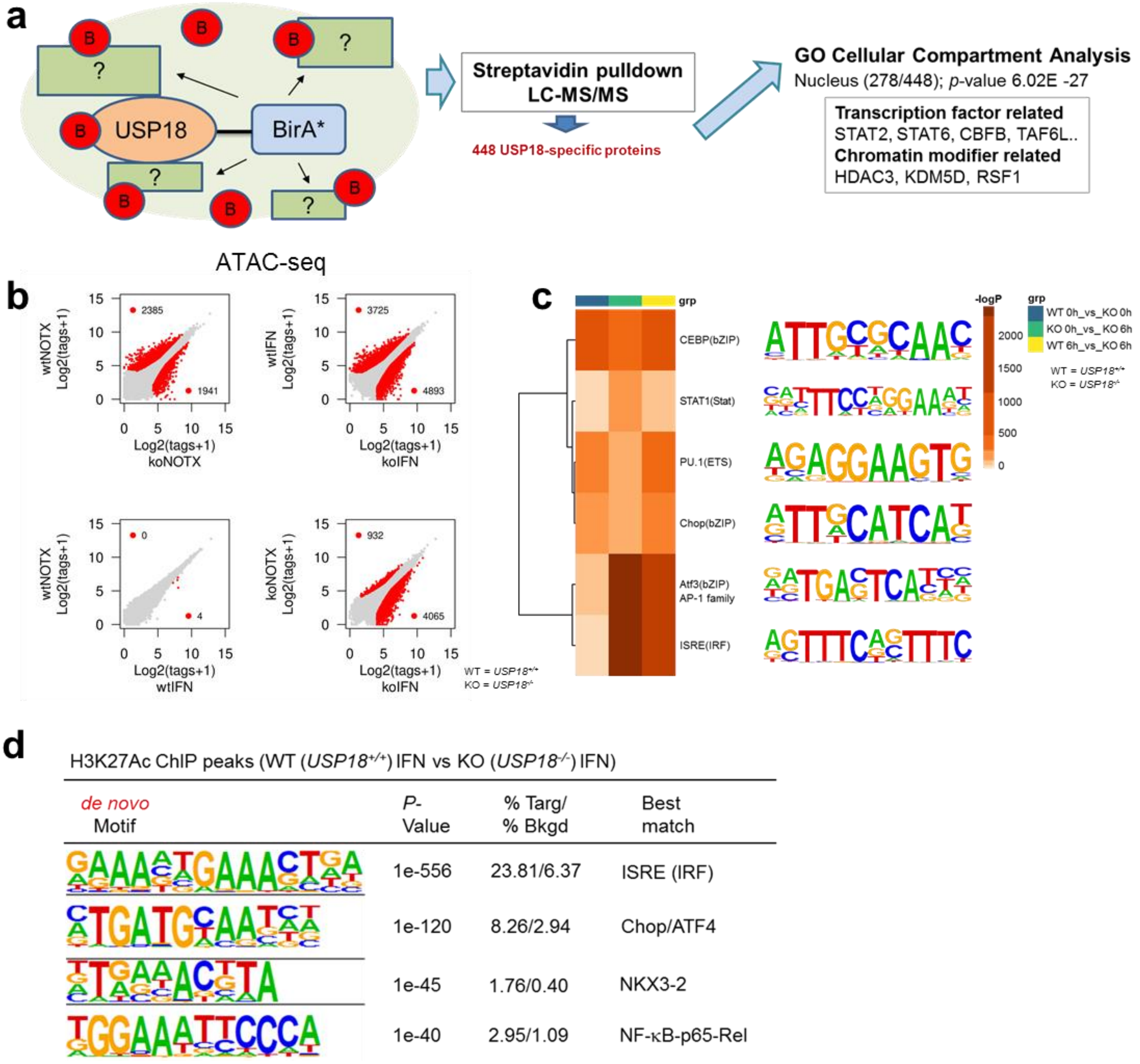

Supplementary Fig. 5

Nuclear USP18 plays a role in ISG expansion

a; Schematic of the strategy for performing proximity-based biotin labeling in the U5A cell line. Three independent replicates were submitted for mass-spectrometric analysis and 408 unique, high-confidence USP18-proximal proteins were identified. Gene Ontology (GO) cellular compartment analysis was performed on this group of proteins.

b; Scatter plot of ATAC tag counts at genomic regions marked by significant ATAC in WT vs *USP18*$^{-/-}$, WT vs WT IFN, WT IFN vs *USP18*$^{-/-}$ IFN, and *USP18*$^{-/-}$ vs *USP18*$^{-/-}$ IFN THP-1

cells. Red data points refer to peaks with >2-fold change.

c; Comparative motif enrichment defined by ATAC-seq in the comparison from (b). *p*-value was determined by two-tailed Hypergeometric test adjusted with Benjamini-Hochberg approach.

d; Top 4 *de novo* motifs at enhancer regions defined by differential H3K27ac (WT IFN vs *USP18*$^{-/-}$ IFN). *p*-value was determined by two-tailed Hypergeometric test adjusted with Benjamini-Hochberg approach.

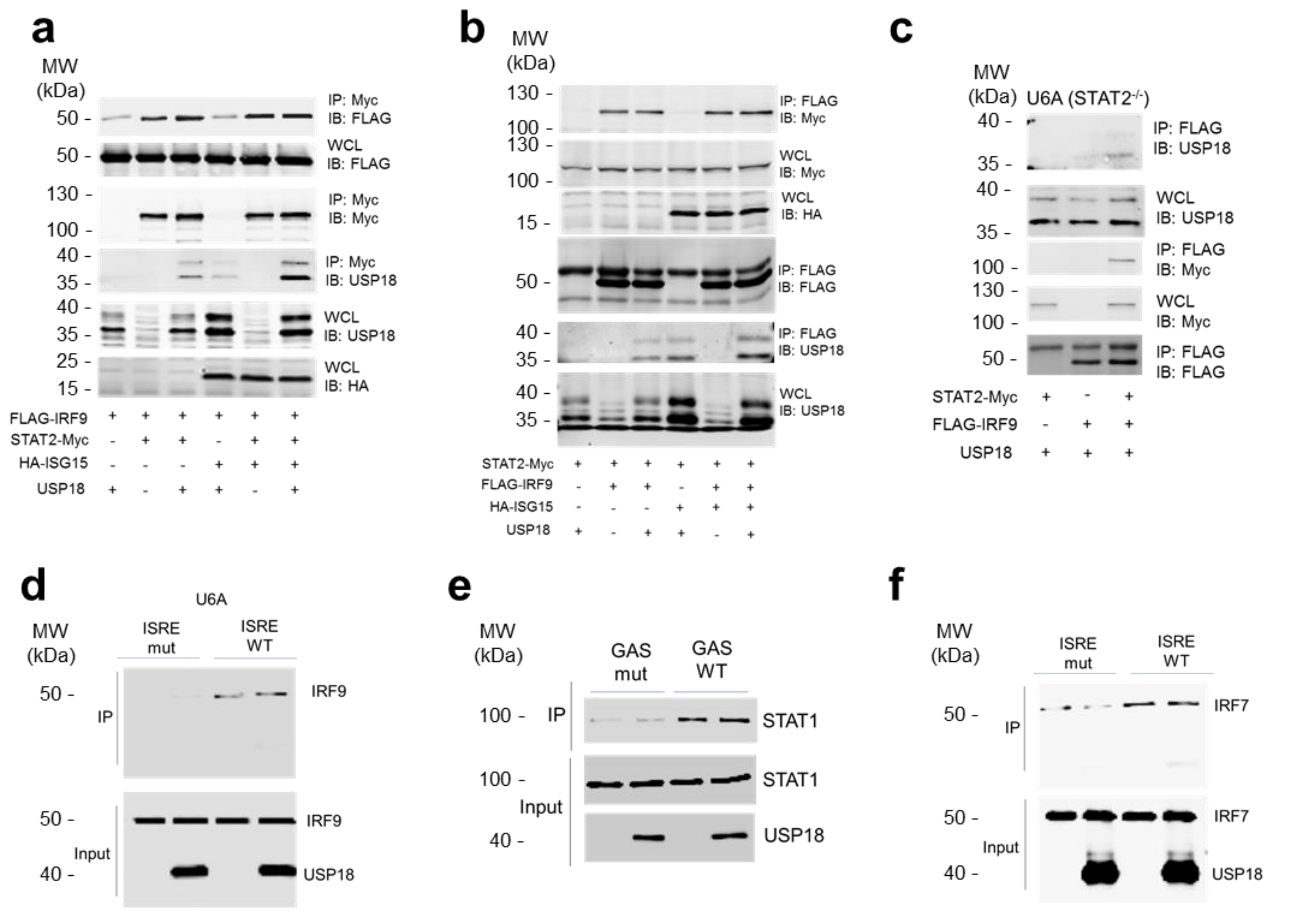

Supplementary Fig. 6

USP18 inhibits ISRE or IRE-IRF9 binding in a STAT2 dependent manner

a; 293T cells were transfected with plasmids encoding FLAG-IRF9, STAT2-Myc, HA-ISG15, and USP18 as indicated. Cell lysates were immunoprecipitated with anti-Myc antibody and immunoblotted with FLAG, Myc, and USP18 antibodies. Whole cell lysates were also analyzed with FLAG, USP18, and HA antibodies.

b; 293T cells were transfected with plasmids encoding FLAG-IRF9, STAT2-Myc, HA-ISG15, and USP18 as indicated. Cell lysates were immunoprecipitated with anti-FLAG antibody and immunoblotted with FLAG, Myc, and USP18 antibodies. Whole cell lysates were also analyzed with Myc, USP18, and HA antibodies.

c; U6A cells were transfected with plasmids encoding FLAG-IRF9, STAT2-Myc, and USP18 as indicated. Cell lysates were immunoprecipitated with anti-FLAG antibody and immunoblotted with FLAG, Myc, and USP18 antibodies. Whole cell lysates were also analyzed with Myc, and USP18 antibodies.

d; DNA pulldown assay in U6A cells. ISRE-IRF9 binding was not affected by USP18 expression.

e; DNA pulldown assay. GAS-STAT1 binding was not affected by USP18 expression.

f; DNA pulldown assay. ISRE-IRF7 binding was not affected by USP18 expression.

Source data are provided as a Source Data file.

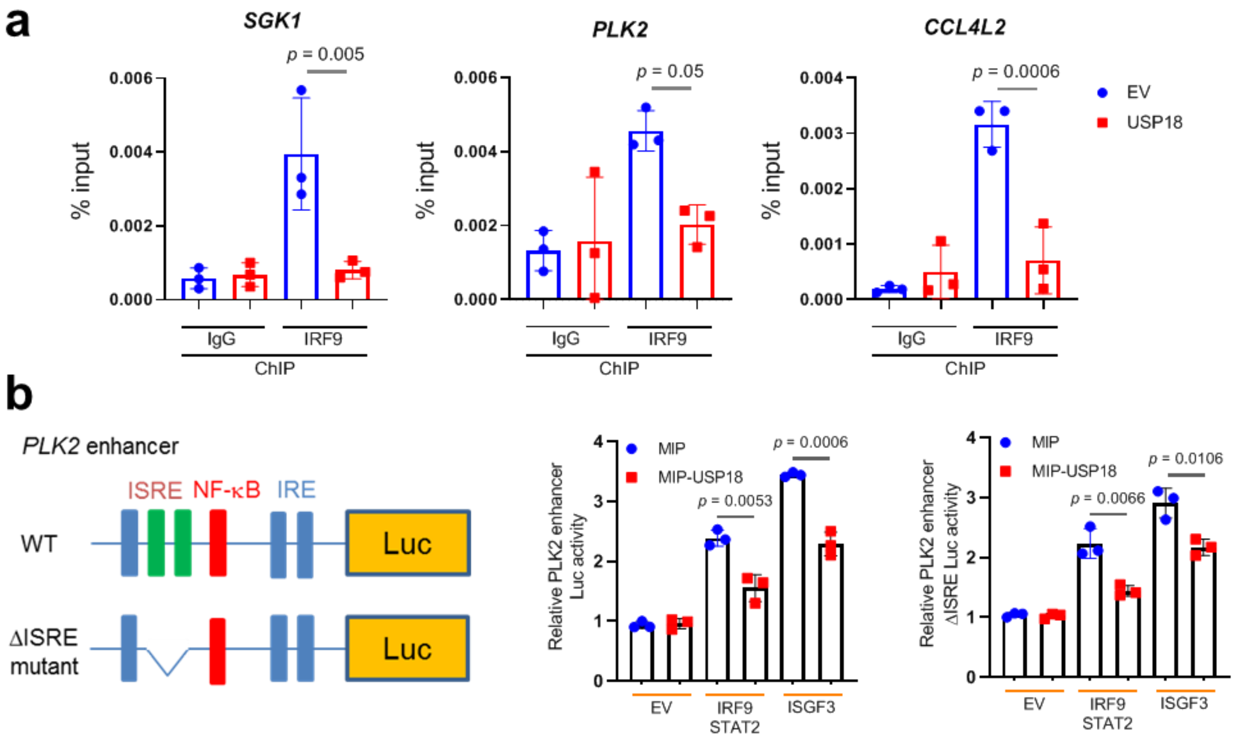

Supplementary Fig. 7

USP18 regulates enhancer activity of hidden atypical ISGs and non-canonical atypical ISGs

a; IRF9 ChIP qPCR for *SGK1*, *PLK2,* and *CCL4L2* in empty vector (EV) or USP18 expressing THP-1 cells. n = 3 independent samples. *p*-value was determined by one-way ANOVA test.

b; Relative firefly to renilla luciferase activity in U5A (*IFNAR2*$^{-/-}$) cells transfected with the indicated luciferase reporter construct with or without USP18 co-expression. n = 3 independent samples. *p*-value was determined by one-way ANOVA test.

All data represent mean ± s.d.

Source data are provided as a Source Data file.

Supplementary Figure 8 (Arimoto et al)

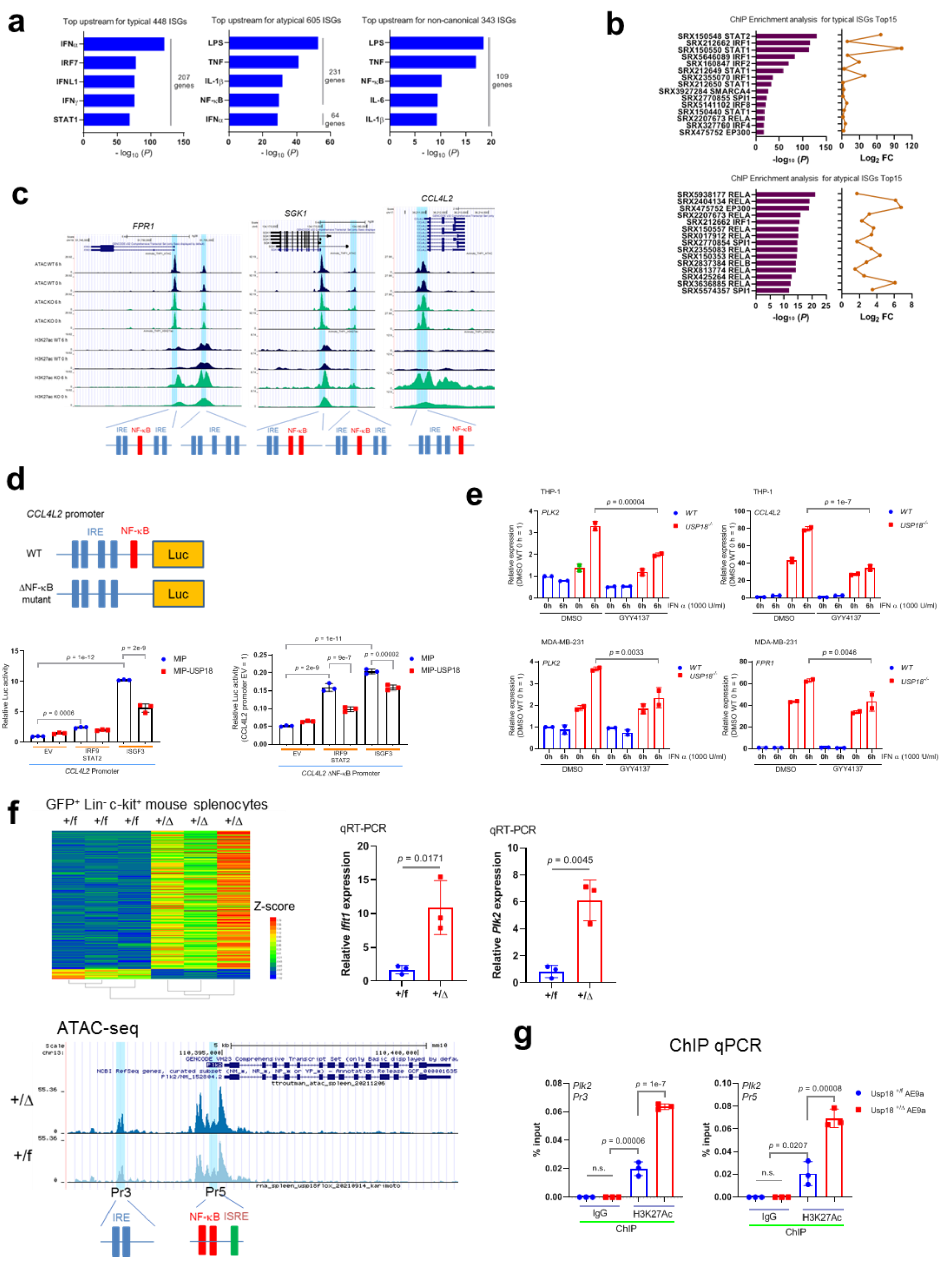

Supplementary Fig. 8

NF-κB (p65) is involved in the induction of atypical ISGs

a; IPA upstream analysis of typical, atypical, and non-canonical ISGs. *p*-value was determined by right-tailed Fisher's exact test adjusted with Benjamini-Hochberg approach.

b; ChIP-Atlas Enrichment analysis for typical and atypical ISGs. *p*-value was determined by two-tailed Fisher's exact probability test adjusted with Benjamini-Hochberg approach.

c; Genome browser tracks depicting ATAC and H3K27ac peaks in *CCL4L2*, *SGK1*, and *FPR1*.

d; Relative firefly to renilla luciferase activity in cells co-transfected with the indicated *CCL4L2* promoter luciferase construct and the indicated combinations of IRF9, STAT2, p65, and USP18. *p*-value was determined by one-way ANOVA test.

e; qRT-PCR analysis of *PLK2* and *CCL4L2* expression in parental or $USP18^{-/-}$ THP-1 cells with the indicated combination of IFN, DMSO, and GYY4137 (100 μM) treatments. Cells were pre-treated with DMSO or GYY4137 2 hours before IFN treatment. *p*-value was determined by one-way ANOVA test.

f; Paired bulk RNA-seq and ATAC-seq data from $GFP^{+}Lin^{-}c\text{-}Kit^{+}$ splenocytes from $Usp18^{+/f}$ and $Usp18^{+/\Delta}$ AE9a recipients (*Top and bottom*). Increased *Ifit1* and *Plk2* expressions in $GFP^{+}Lin^{-}c\text{-}Kit^{+}$ splenocytes from $Usp18^{+/\Delta}$ AE9a recipients were also validated by qRT-PCR (*Top Right*). *p*-value was determined by two-tailed Student *t*-test.

g; H3K27ac ChIP qPCR was performed with primers in promoter/enhancer locus of *Plk2* in $GFP^{+}Lin^{-}c\text{-}Kit^{+}$ splenocytes from $Usp18^{+/f}$ and $Usp18^{+/\Delta}$ AE9a recipients. *p*-value was determined by one-way ANOVA test. All data represent mean ± s.d, except where indicated. n.s. = not statistically significant. Source data are provided as a Source Data file.

Supplementary Figure 9 (Arimoto et al)

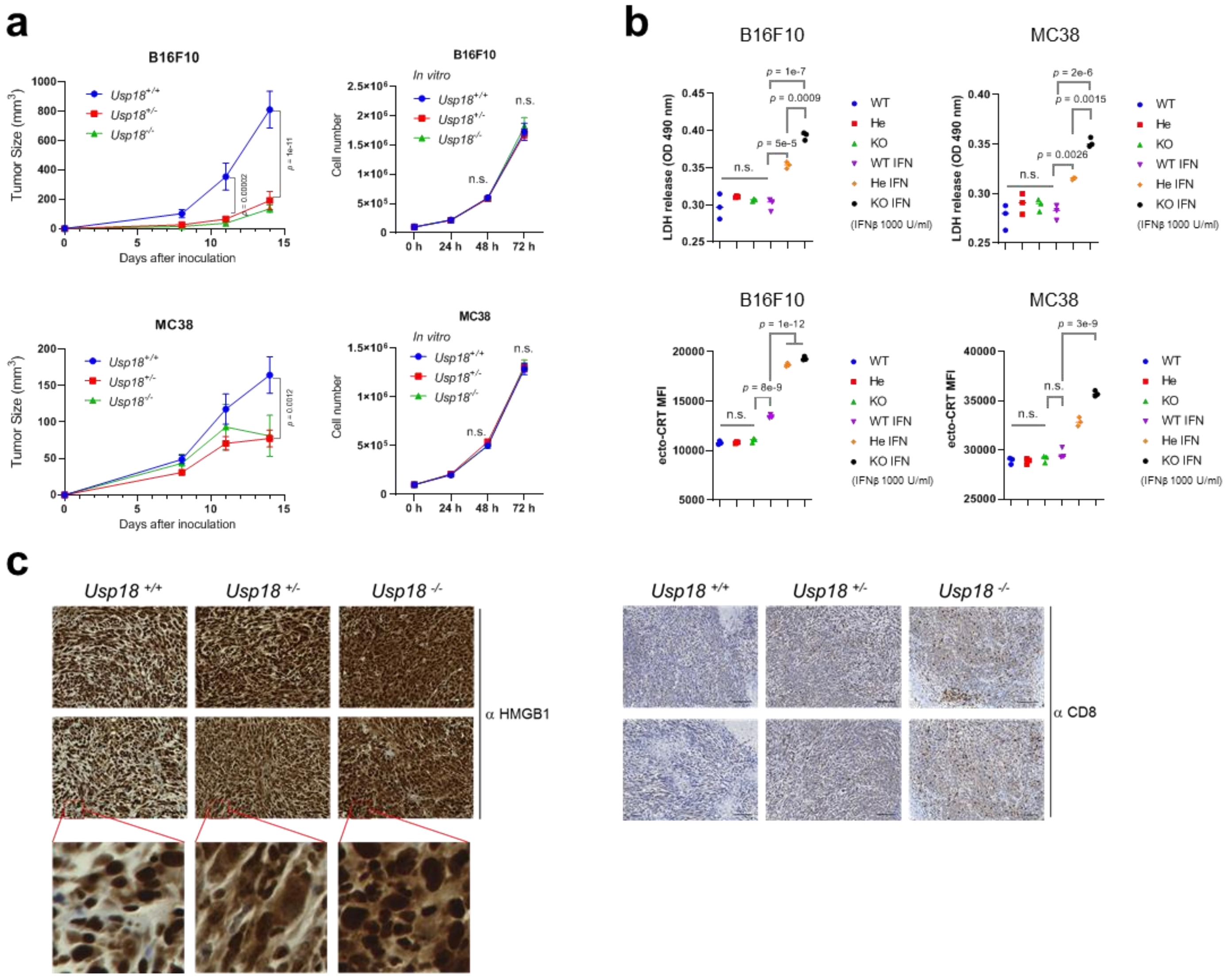

Supplementary Fig. 9

USP18 loss delay tumorigenesis and induce ICD in solid cancer mouse models

a; Tumor volume derived from subcutaneous injection (s.c.) of *USP18*$^{+/+}$ (n = 5), *USP18*$^{+/-}$ (n = 5), or *USP18*$^{-/-}$ (n = 5) B16F10 or *USP18*$^{+/+}$ (n = 6), *USP18*$^{+/-}$ (n = 5), or *USP18*$^{-/-}$ (n = 5) MC38 cells into C57/BL6 mice. Tumor volume were calculated from the length (a) and width (b) by using the following formula; volume (mm^3) = $ab^2/2$. Data are presented as mean values ±SEM. *p*-value was determined by two-way ANOVA test (*left*). Growths of these cells *in vitro* are also shown (*right*) (n = 3 independent samples each). n.s. = not statistically significant. b; LDH release assay and MFI of surface-exposed calreticulin (ecto-CRT) of *USP18*$^{+/+}$, *USP18*$^{+/-}$, or *USP18*$^{-/-}$ B16F10 or MC38 cells treated with or without murine IFNβ (1000U/ml) for 48 hours. (n = 3 independent samples each). *p*-value was determined by one-way ANOVA test. Data are represented as mean ± s.d, except where indicated. n.s. = not

statistically significant.

c; Immunohistochemistry analysis of tumors from *USP18*$^{+/+}$, *USP18*$^{+/-}$, or *USP18*$^{-/-}$ MC38 cells injected mice stained with either anti HMGB1 or CD8 antibody and nuclear hematoxylin staining (blue) (HMGB1; Bar = 50 μm, CD8; Bar = 100 μm).

Source data are provided as a Source Data file.

Supplementary Figure 10 (Arimoto et al)

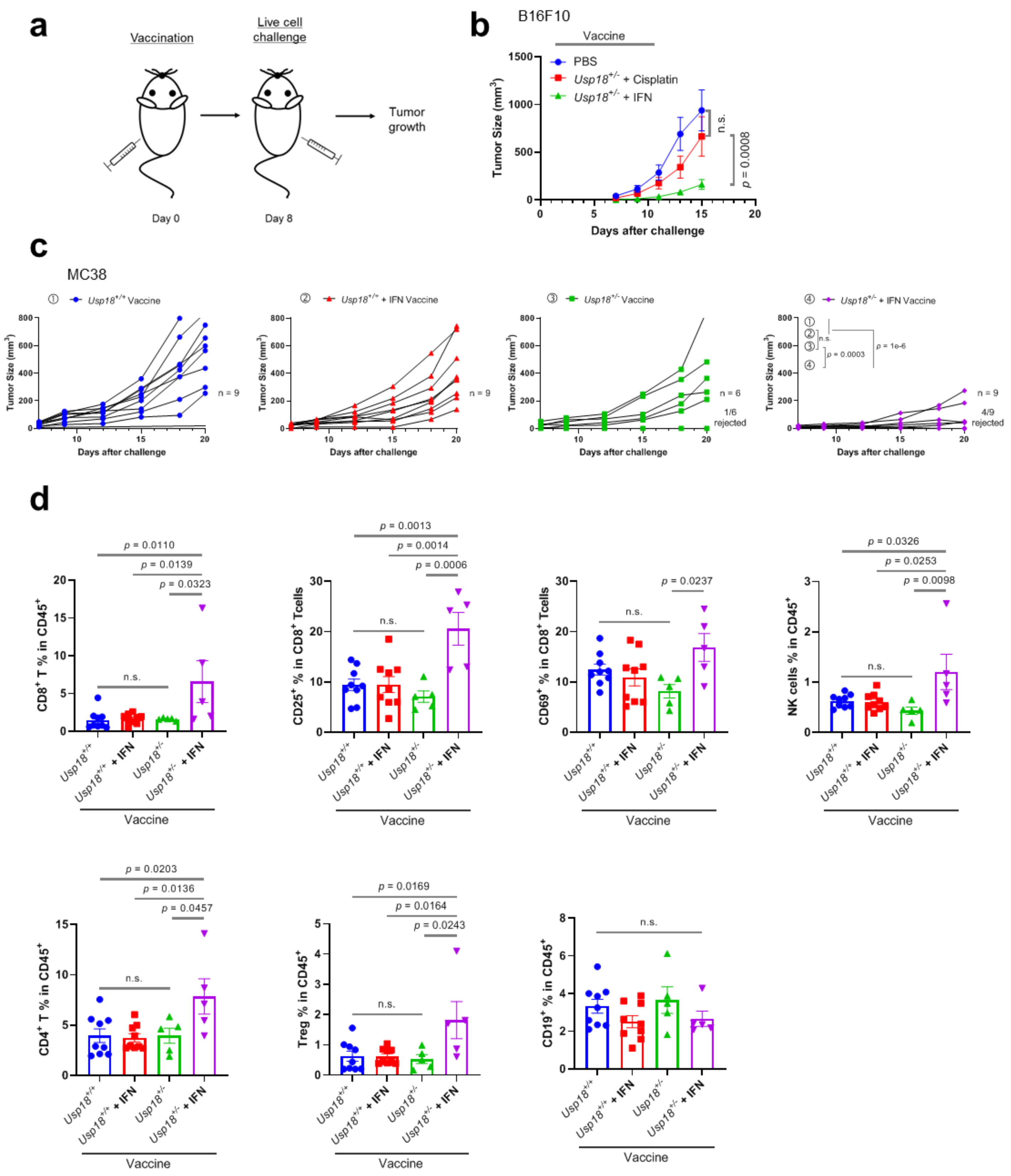

Supplementary Fig. 10

IFN treated USP18 depleted cancer cell vaccine induces anti-tumor response

a; A schematic depiction of the in vivo vaccination assay treatment schedule for assaying immunogenic cell death.

b; Tumor volume resulting from mice vaccinated with dying *USP18*$^{+/-}$ B16F10 cells via cisplatin or IFNβ treatment and subsequently challenged with live B16F10 murine melanoma cells. PBS injection was also done for control vaccination. PBS (n = 6), *USP18*$^{+/-}$ B16F10 + cisplatin (n = 7), *USP18*$^{+/-}$ B16F10 + IFNβ (n = 7). Data are presented as mean values ±SEM. *p*-value was determined by two-way ANOVA test.

c; Tumor volume resulting from mice vaccinated with or without IFNβ treated *USP18*$^{+/+}$ or *USP18*$^{+/-}$ MC38 cells were subsequently challenged with live MC38 murine colon cancer cells. Tumor growth from all mice are shown. *USP18*$^{+/+}$ (n = 9), *USP18*$^{+/+}$ + IFNβ (n = 9), *USP18*$^{+/-}$ (n = 6), *USP18*$^{+/-}$ + IFNβ (n = 9). *p*-value was determined by two-way ANOVA test.

d; Lymphocytes infiltration in challenged tumors of (c) was analyzed by FACS. NK ($CD3^-$ $NK1.1^+$), Treg ($CD4^+CD25^+Foxp3^+$). *p*-value was determined by one-way ANOVA test.

Data are represented as mean ± s.d, except where indicated. n.s. = not statistically significant.

Source data are provided as a Source Data file.

Supplementary Figure 11 (Arimoto et al)

a

Commonly upregulated genes in human and mouse RNAseq	IFN inducible	apoptosis related
IRF7	〇	×
CD7	〇	×
RTP4	〇	×
SLFN5	〇	×
DHX58	〇	×
IFIT1	〇	〇
CDKN1A	〇	〇
PLK2	×	〇

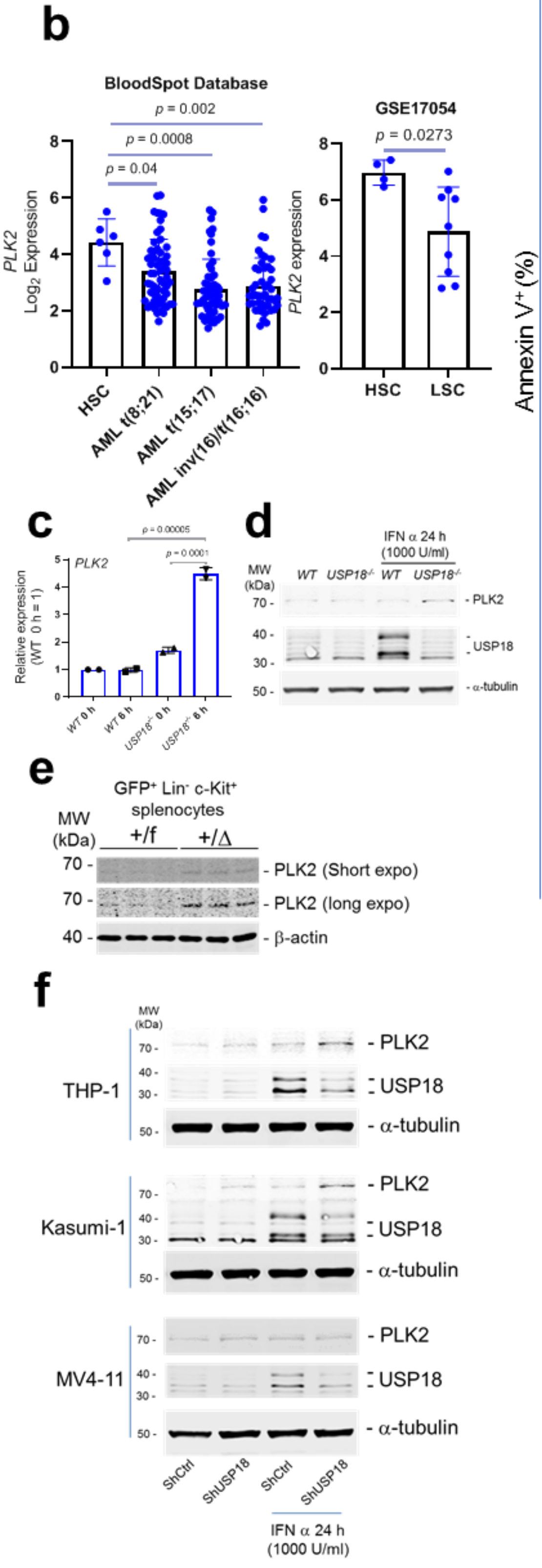

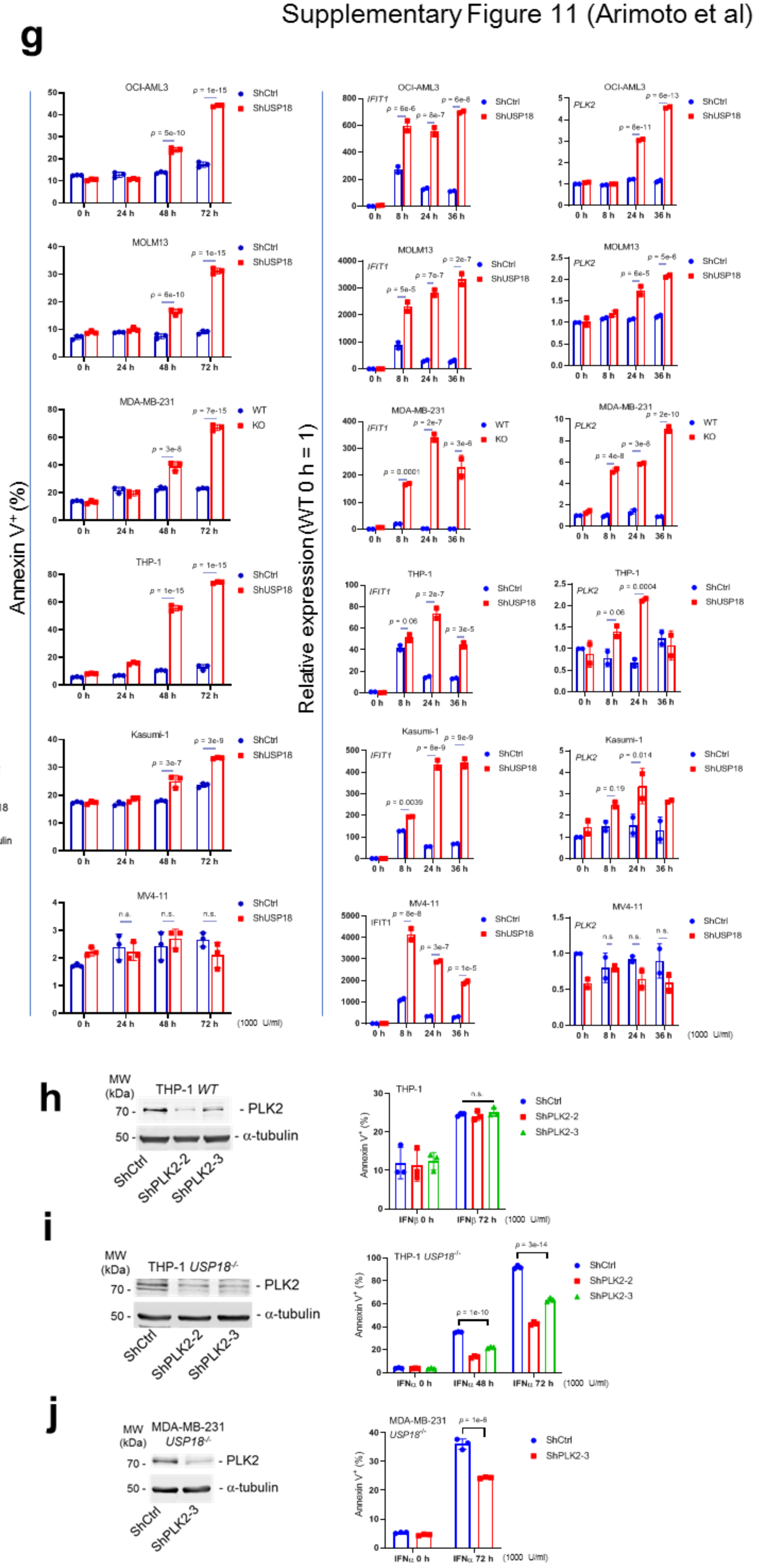

Supplementary Fig. 11

PLK2 is downregulated in human AML patients and is one of the critical atypical ISGs that contributes to IFN treated USP18KD cell lethality

a; Genes commonly upregulated (RNA-seq analysis) in *USP18*$^{-/-}$ + IFN human cell lines and *Usp18* knockdown murine AML cells.

b; Expression analyses of *PLK2* in healthy human hematopoietic stem cells (HSC) and AML patients (left) (n = 6, 60, 54, 47) or leukemic stem cells (n =4, 9) (LSC) (right).

c; qRT-PCR analysis for *PLK2* in WT and *USP18*$^{-/-}$ THP-1 cells with or without IFNα (1000U/ml) treatment for 6 h (n = 3 independent samples each).

d; Western blot analysis of WT and *USP18*$^{-/-}$ THP-1 cells were treated with or without IFN for 36 hours.

e; Western blot analysis of sorted GFP^{+}Lin^{-}c-Kit^{+} splenocytes from *Usp18*$^{+/f}$ and *Usp18*$^{+/\Delta}$ AE9a recipient mice (n = 3 each).

f; Western blot analysis of ShCtrl or ShUSP18 expressing THP-1, Kasumi-1, and MV4-11 cells were treated with IFNα (1000U/ml) for 24 hours.

g; FACS analysis of ShCtrl or ShUSP18 expressing OCI-AML3, MOLM13, MDA-MB-231, THP-1, Kasumi-1, and MV4-11 cells treated with IFNα (1000U/ml) for the indicated times. The same cells were treated with IFN for the indicated times. *IFIT1* and *PLK2* expression were analyzed by qRT-PCR (n = 3 independent samples each).

h; PLK2 expression of THP-1 WT cells infected with ShCtrl, ShPLK2-2, and, ShPLK2-3 lentiviruses (*Left*). The percentage of Annexin V^{+} cells was also measured in these cells after IFNβ (1000U/ml) treatment for the indicated times (n = 3 independent samples each) (*Right*).

i; PLK2 expression of *USP18*$^{-/-}$ THP-1 cells infected with ShCtrl, ShPLK2-2, and, ShPLK2-3 lentiviruses (*Left*). The percentage of Annexin V^{+} cells was also measured in these cells after IFNα (1000U/ml) treatment for the indicated times (n = 3 independent samples each) (*Right*).

j; PLK2 expression of *USP18*$^{-/-}$ MDA-MB-231 cells infected with ShCtrl and ShPLK2-3 lentiviruses (*Left*) and percentage of annexin V^{+} cells after IFNα (1000U/ml) treatment was analyzed by FACS (n = 3 independent samples each) (*Right*). All *p*-values in Supplementary Fig.11 were determined by one-way ANOVA test, except for 11b right (two-tailed Student *t*-test). All data represent mean ± s.d, except where indicated. n.s. = not statistically significant. Source data are provided as a Source Data file.

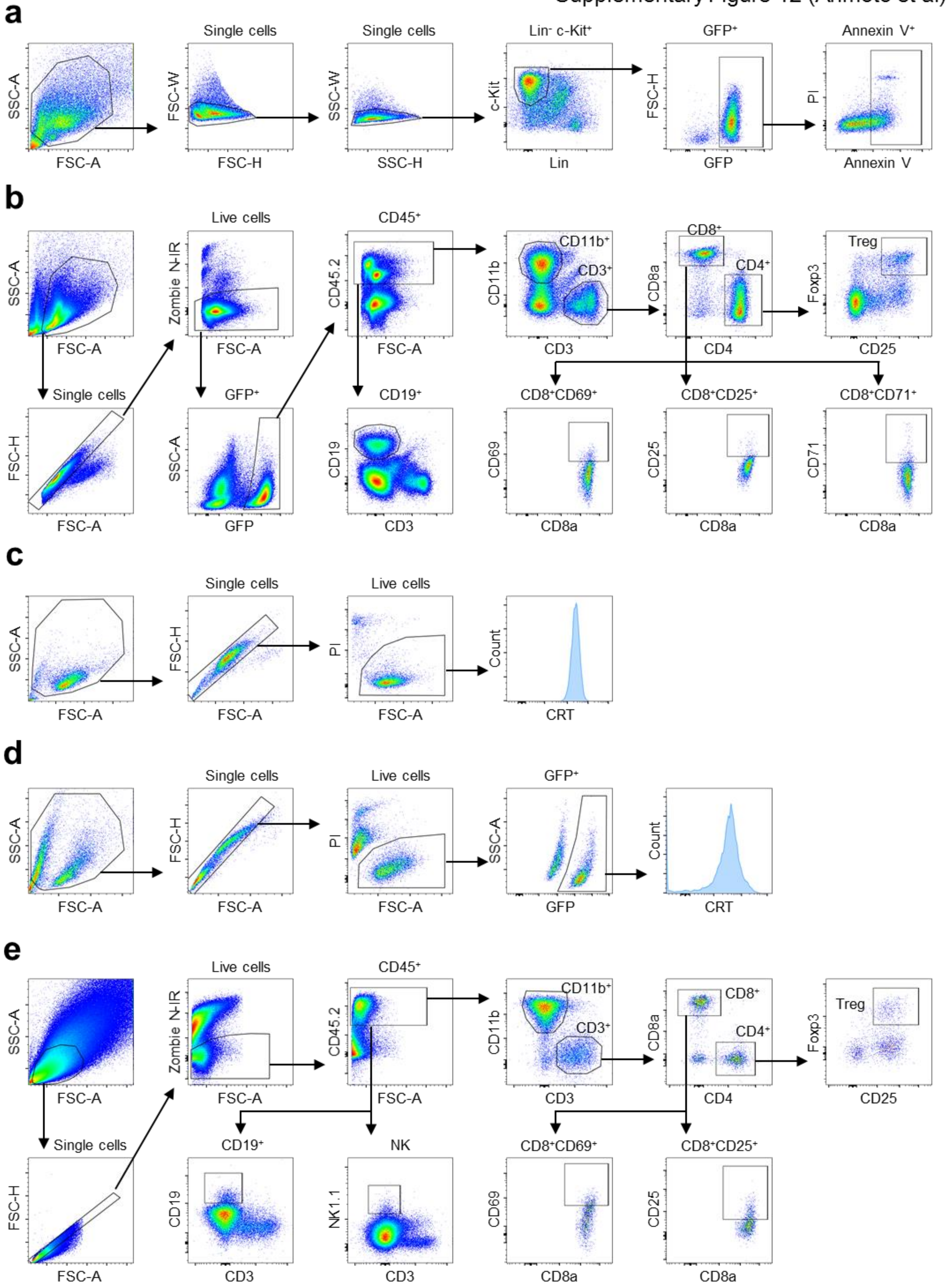
a
Single cells
Single cells
Lin- c-Kit+
GFP+
Annexin V+
SSC-A
FSC-A
FSC-W
FSC-H
SSC-W
SSC-H
c-Kit
Lin
FSC-H
GFP
PI
Annexin V
b
Live cells
CD45+
CD11b+
CD3+
CD8+
CD4+
Treg
SSC-A
FSC-A
Zombie N-IR
FSC-A
CD45.2
FSC-A
CD11b
CD3
CD8a
CD4
Foxp3
CD25
Single cells
GFP+
CD19+
CD8+CD69+
CD8+CD25+
CD8+CD71+
FSC-H
FSC-A
SSC-A
GFP
CD19
CD3
CD69
CD8a
CD25
CD8a
CD71
CD8a
c
Single cells
Live cells
SSC-A
FSC-A
FSC-H
FSC-A
PI
FSC-A
Count
CRT
d
Single cells
Live cells
GFP+
SSC-A
FSC-A
FSC-H
FSC-A
PI
FSC-A
SSC-A
GFP
Count
CRT
e
Live cells
CD45+
CD11b+
CD3+
CD8+
CD4+
Treg
SSC-A
FSC-A
Zombie N-IR
FSC-A
CD45.2
FSC-A
CD11b
CD3
CD8a
CD4
Foxp3
CD25
Single cells
CD19+
NK
CD8+CD69+
CD8+CD25+
FSC-H
FSC-A
CD19
CD3
NK1.1
CD3
CD69
CD8a
CD25
CD8a

Supplementary Fig. 12

Gating strategies in this study.

a; Gating strategy to analyze Annexin V$^+$ cells in GFP$^+$Lin$^-$c-Kit$^+$ *Usp18*$^{+/\Delta}$ leukemia cells; associated with Fig 1d.

b; Gating strategy to analyze immune populations in viable splenocytes in recipients of *Usp18*$^{+/\Delta}$ AML cells; associated with Fig 1e.

c; Gating strategy to analyze ecto-CRT in cell lines; associated with Fig 6d, 7b, 7l, 7o, 7t, 8c, 8f and Supplementary Fig 9b.

d; Gating strategy to analyze ecto-CRT in AML cells from mice; associated with Fig 6j

e; Gating strategy to analyze tumor immune filtrating cells to evaluate vaccine effect of dying *Usp18*$^{+/-}$ cells; associated with Supplementary Fig 10d.

Table 1 : Genotype analysis of *USP18* heterozygous intercross progeny.

	Genotype			
	Usp18$^{+/+}$	*Usp18* $^{+/-}$	*Usp18* $^{-/-}$	
			Alive	Dead
E12.5	14	24	14	0
E13.5	13	20	6	0
E14.5	6	15	1* * dying	6

Ifnar1$^{-/-}$ *Usp18*$^{-/-}$; All alive

Supplementary Table 1

Genotype analysis of *Usp18* heterozygous intercross progeny.

Usp18$^{-/-}$ are embryonic lethal.

Table 2. Embryonic lethality of USP18 knockout mice is due to the blood system problem

Usp18 $^{+/f}$ *CMV-Cre* x *Usp18* $^{-/f}$		(n = 43)	*Usp18* $^{+/f}$ *Vav-iCre* x *Usp18* $^{-/f}$		(n = 34)	*Usp18* $^{+/f}$ *UBC^{ER}-Cre* x *Usp18* $^{-/f}$		(n = 32)
CMV-Cre -			*Vav-iCre* -			*UBC^{ER}-Cre* -		
	f/f	9		f/f	4		f/f	4
	-/f	4		-/f	2		-/f	6
	+/f	6		+/f	9		+/f	3
	+/-	7		+/-	6		+/-	3
CMV-Cre +			*Vav-iCre* +			*UBC^{ER}-Cre* +		
	f/f	0		f/f	0		f/f	7
	-/f	0		-/f	0		-/f	3
	+/f	8		+/f	5		+/f	1
	+/-	9		+/-	8		+/-	5

Supplementary Table 2

Embryonic lethality of *Usp18* deficient mice is due to the blood system problem.

Survival of *Usp18* $^{f/f,\ -/f,\ +/f,\ \mathrm{or}\ +/-}$ *CMV-Cre* $^{-\ \mathrm{or}\ +}$ (n = 43), *Vav-iCre* $^{-\ \mathrm{or}\ +}$ (n = 34), and *UBC^{ER}-Cre* $^{-\ \mathrm{or}\ +}$ (n = 32) mice were examined.

Table 3. Usp18 depletion in mice leads to leukopenia like phenotype

Hematological Values	*Usp18*$^{f/f}$	*Usp18*$^{\Delta/\Delta}$	*Usp18*$^{-/f}$	*Usp18*$^{-/\Delta}$
WBC (x 10^3/μl)	6.5±1.2	1.6±0.4	8.5±1.2	2.7±1.7
NE (x 10^3/μl)	1.5±0.2	0.4±0.0	2.0±0.1	0.7±0.4
LY (x 10^3/μl)	4.7±1.1	1.0±0.4	6.1±1.3	1.4±1.6
MO (x 10^3/μl)	0.3±0.2	0.1±0.0	0.3±0.0	0.1±0.1
EO (x 10^3/μl)	0.03±0.02	0.04±0.04	0.11±0.07	0.09±0.06
BA (x 10^3/μl)	0.01±0.01	0.01±0.01	0.01±0.01	0.01±0.00
RBC(x 10^6/μl)	9.7±0.8	10.0±1.2	9.3±0.4	9.5±0.4
HB (g/dL)	12.0±0.7	13.6±1.7	11.6±0.9	11.6±0.7
HCT (%)	48.3±5.0	55.3±8.0	44.8±1.3	45.9±2.7
MCV (fl)	49.5±1.7	50.8±2.1	48.4±0.8	48.5±0.9
MCH (pg)	12.3±0.6	12.5±0.4	12.5±1.4	12.3±1.2
MCHC (%)	24.9±1.2	24.6±0.6	25.8±2.5	25.5±3.0
PLT (x 10^3/μl)	2336.3±171.8	1462±323.7	1811.0±363.4	1473.0±432.4
n	3	3	3	3

WBC (x 10^3/ μ l) : White blood cells
NE (x 10^3/ μ l) : Neutrophils
LY (x 10^3/ μ l) : Lymphocytes
MO (x 10^3/ μ l) : Monocytes
EO (x 10^3/ μ l) : Eosinophils
BA (x 10^3/ μ l) : Basophils
RBC (x 10^6/ μ l) : Red blood cells
HB (g/dL) : Hemoglobin
HCT (%) : Hematocrit
MCV (fl) : Mean corpuscular volume
MCH (pg) : Mean corpuscular hemoglobin
MCHC (%) : Mean corpuscular hemoglobin concentration
PLT (x 10^3/ μ l) : Platelets

Supplementary Table 3

***Usp18* deletion in mice leads to leukopenia like phenotype.**

Peripheral blood counts were analyzed at day 5 for *Usp18*$^{f/f}$, *Usp18*$^{f/f}$ *UBC*^{ER}*-Cre*, *Usp18*$^{-/f}$ *UBC*^{ER}*-Cre*, and *Usp18*$^{-/f}$ *UBC*^{ER}*-Cre* mice after 3 consecutive times tamoxifen injection. Data are the mean ± s.d.; n=3 per genotype.

Table 4. Guide RNA sequence primers

USP18 gRNA

Primers	Sequence
gRNA set1	F:5'-CACCGAAGTCGTGCTGTCCTGAACG-3'
	R:5'-AAACCGTTCAGGACAGCACGACTTC-3'
gRNA set2	F:5'-CACCGAGCAGCCCAGAGAGCGTCCC-3'
	R:5'-AAACGGGACGCTCTCTGGGCTGCTC-3'

mUsp18 gRNA

Primers	Sequence
gRNA set1	F:5'-CACCGTGCCAGAGGACGACTGGTTA-3'
	F:5'-AAACTAACCAGTCGTCCTCTGGCAC-3'
gRNA set2	F:5'-CACCGGCCGCAGCAGTACTCAGCGC-3'
	F:5'-AAACGCGCTGAGTACTGCTGCGGCC-3'

PLK2 sgRNA

Primers	Sequence
gRNA set	F:5'-CACCGACGATAATCCGCGAGATCTC-3'
	F:5'-AAACGAGATCTCGCGGATTATCGTC-3'

GSDMD sgRNA

Primers	Sequence
gRNA set1	F:5'-CACCGTGGGTCCCTGCACCACGCTC-3'
	F:5'-AAACGAGCGTGGTGCAGGGACCCAC-3'
gRNA set2	F:5'-CACCGGAGCCGGATGCCGCGGAACC-3'
	F:5'-AAACGGTTCCGCGGCATCCGGCTCC-3'

GSDME sgRNA

Primers	Sequence
gRNA set	F:5'-CACCGGAAGACCAATTTCCGAGTCC-3'
	F:5'-AAACGGACTCGGAAATTGGTCTTCC-3'

Supplementary Table 4

Guide RNA sequence primers

In formation for guide RNA sequences for Crispr in this study.

Table 5. RT-qPCR primers

RT qPCR primers 5'-3' for murine genes

Primers	Sequence
Isg15	F:5'-GACTAACTCCATGACGGTG-3'
	R:5'-AACTGGTCTTCGTGACTTG-3'
Ifit1	F:5'-TGGCGACCTGGGGCAACTGTG-3'
	R:5'-TGGGCTGCCTGTTTCGGGATGTC-3'
Irf7	F:5'-CAGCGAGTGCTGTTTGGAGAC-3'
	R:5'-AAGTTCGTACACCTTATGCGG-3'
Oasl2	F:5'-TTGTGCGGAGGATCAGGTACT-3'
	R:5'-TGATGGTGTCGCAGTCTTTGA-3'
Dhx58	F:5'-CCAGAAAGACCAGCAGGAAG-3'
	R:5'-TTGCACTGAGCGATATCCAG-3'
Rtp4	F:5'-GGAGCCTGCATTTGGATAAG-3'
	R:5'-TTCTGCAGCATCTGGAACAC-3'
Xaf1	F:5'-GCCTGCGCTTCATAGTCCTTT-3'
	R:5'-GGTGCACAACTTCCATGTGCT-3'
Plk2	F:5'-CATCACCACCATTCCCACT-3'
	R:5'-TCGTAACACTTTGCAAATCCA-3'
Cd80	F:5'-ACCCCCAACATAACTGAGTCT-3'
	R:5'-TTCCAACCAAGAGAAGCGAGG-3'
Cdkn1a	F:5'-CTGGGAGGGGACAAGAG-3'
	R:5'-GCTTGGAGTGATAGAAATCTG-3'
Zmat3	F:5'-GACTCGGAAAGAAGGGAGTG-3'
	R:5'-AGTAAGGGCCTGAATTGCTC-3'
Eda2r	F:5'-CACACTGCATAGTCTGCCCTC-3'
	R:5'-GCCTTCTGGACCCGATTGA-3'
Tnfsf4	F:5'-GTTCTGCACCTCCATAGTTTGA-3'
	R:5'-GGATGCTTCTGTGCTTCATCT-3'
Slfn5	F:5'-TTCTGCTGTGCGGTGTTTGCCA-3'
	R:5'-CTGGAGAACCATCTCAGGACAC-3'
Cxcl9	F:5'-TCCTTTTGGGCATCATCTTCC-3'
	R:5'-TTTGTAGTGGATCGTGCCTCG-3'
Gapdh	F:5'-AAGGTCATCCCAGAGCTGAA-3'
	R:5'-CTGCTTCACCACCTTCTTGA-3'

RT qPCR primers 5'-3' for human genes

Primers	Sequence
IFIT1	F:5'-AAGGCAGGCTGTCCGCTTA-3'
	R:5'-TCCTGTCCTTCATCCTGAAGCT-3'
PLK2	F:5'-GACCCTATGGGACTCCTCTTT-3'
	R:5'-GTATGCCTTAGCCTGTTCTGG-3'
FPR1	F:5'-CCTCCACTTTGCCATTC-3'
	R:5'-AGCAGAGCCATCACCC-3'
SGK1	F:5'-GACAGGACTGTGGACTGGTG-3'
	R:5'-TTTCAGCTGTGTTTCGGCTA-3'
CCL4L2	F:5'-AAAACCTCTTTGCCACCAATACC-3'
	R:5'-GAGAGCAGAAGGCAGCTACTAG-3'
GAPDH	F:5'-GCCAAAAGGGTCATCATCT-3'
	R:5'-ATGGATGACCTTGGCCAG-3'

Supplementary Table 5

RT-qPCR primers

Information for RT-qPCR primers in this study.

Table 6. Primers for ChIP-qPCR

IRF9 ChIP qPCR

Primers	Sequence
SGK1	F:5'-TGAGAATTGCCACCATGCCCCTCAT-3'
	R:5'-AGCATACGCCGAGCCGGTCTTTGA-3'
PLK2	F:5'-GTGTCTCAAATACTTCCAGCA-3'
	R:5'-TGGACTTGCCCTGCACTCTTTA-3'
CCL4L2	F:5'-AAGCCACTTGTAGCAGGTGT-3'
	R:5'-CAGGGCGATGTCATCATA-3'

p65 ChIP qPCR

Primers	Sequence
SGK1	F:5'-TCAAAGACCGGCTCGGCGTAT-3'
	R:5'-AAGGGCGGGGCCACTTCTCACTGT-3'
PLK2	F:5'-GTCTTTGCTGTCACTTCCGT-3'
	R:5'-CTTATTCCATCCCTGACAATCT-3'
CCL4L2	F:5'-ACTCCTCACTCAGCTAGCTT-3'
	R:5'-CTCTGAGGCCTAGGACAAAT-3'

H3K27ac ChIP qPCR

Primers	Sequence
Plk2 Pr3	F:5'-ATTGGGATTCGGGCACTAAAGTC-3'
	F:5'-GTTCTACCAAAAAGATACAGGC-3'
Plk2 Pr5	F:5'-TTCCGCAAGGCCGGCCACTGA-3'
	F:5'-TGCCCTGACTTTGGACGT-3'

Supplementary Table 6

Primers for ChIP-qPCR

Information for ChIP-qPCR primers in this study.
