# Supplementary material for: Expansion of interferon inducible gene pool via USP18 inhibition promotes cancer cell pyroptosis
Source: Nat Commun. 2023 Jan 17;14:251. doi: 10.1038/s41467-022-35348-5 (PMC9842760; doi:10.1038/s41467-022-35348-5)
Supplement: Supplementary file 3 — Reporting Summary [file 41467_2022_35348_MOESM3_ESM.pdf]

Corresponding author(s): Dong-Er Zhang
Last updated by author(s): Nov 14, 2022

nature research

Reporting Summary

Nature Research wishes to improve the reproducibility of the work that we publish. This form provides structure for consistency and transparency in reporting. For further information on Nature Research policies, see our Editorial Policies and the Editorial Policy Checklist.

Statistics

For all statistical analyses, confirm that the following items are present in the figure legend, table legend, main text, or Methods section.

n/a	Confirmed	
☐	☒	The exact sample size (*n*) for each experimental group/condition, given as a discrete number and unit of measurement
☐	☒	A statement on whether measurements were taken from distinct samples or whether the same sample was measured repeatedly
☐	☒	The statistical test(s) used AND whether they are one- or two-sided<br>*Only common tests should be described solely by name; describe more complex techniques in the Methods section.*
☒	☐	A description of all covariates tested
☐	☒	A description of any assumptions or corrections, such as tests of normality and adjustment for multiple comparisons
☐	☒	A full description of the statistical parameters including central tendency (e.g. means) or other basic estimates (e.g. regression coefficient) AND variation (e.g. standard deviation) or associated estimates of uncertainty (e.g. confidence intervals)
☐	☒	For null hypothesis testing, the test statistic (e.g. *F*, *t*, *r*) with confidence intervals, effect sizes, degrees of freedom and *P* value noted<br>*Give P values as exact values whenever suitable.*
☒	☐	For Bayesian analysis, information on the choice of priors and Markov chain Monte Carlo settings
☒	☐	For hierarchical and complex designs, identification of the appropriate level for tests and full reporting of outcomes
☐	☒	Estimates of effect sizes (e.g. Cohen's *d*, Pearson's *r*), indicating how they were calculated

Our web collection on statistics for biologists contains articles on many of the points above.

Software and code

Policy information about availability of computer code

Data collection	Bulk RNA sequencing data were collected with Illumina Novaseq 6000 (PE150).ATAC sequencing data were collected with Illumina HiSeq 4000. ChIP sequencing data were collected with Illumina Novaseq 6000 (PE150). Single cell RNA sequencing data were collected with Illumina Hiseq 4000. Flow cytometric analysis was performed on a BD FACSCanto with standard lasers and optical filters. Cell sorting was performed using a BD FACSAria-II. RT-qPCR data were collected with CFX96 thermal cycler (BIO-RAD).
Data analysis	The data for bulk RNA-seq were analyzed using the RNASTAR/DeSeq2 pipeline. For ATAC-seq analysis, FASTQ files from ATAC-seq experiments were mapped to the human hg38 genome using Bowtie2 (PMCID: PMC3436841) with default parameters. HOMER software (PMCID: PMC2898526) was used for further analysis. First, peaks were identified using findPeaks with the selected options: 'style factor -minDist 200 -size 200'. Next, peaks were merged using mergePeaks and signals were quantified using annotatePeaks.pl for each data replicate. Peaks within 3000 bp of the transcription start site were excluded, then differentially accessible regions were identified using DeSeq2 (PMCID: PMC4302049) through HOMER getDiffExpression.pl default parameters (fold change > 2 and adjusted p-value < 0.05). For ChIP-seq analysis, The FASTQ files from ChIP-seq experiments were mapped to the human hg38 genome using Bowtie2 with default parameters. HOMER software was used for further analysis. First, pooled ATAC-seq peaks were sorted into promoter proximal or promoter distal peaks using a cutoff of within 3000 bp of the transcription start site. Then H3K27ac ChIP-seq data was quantified at each peak using annotatePeaks.pl within a 1000 bp window of the ATAC-seq peak, as described previously (PMCID: PMC7305990). Differentially acetylated regions were identified using DeSeq2 through HOMER getDiffExpression.pl default parameters (fold change > 2 and adjusted p-value < 0.05). Next, peak windows were shifted back to the original size of the ATAC-seq data and findMotifsGenome.pl was used to assess enrichment of known DNA motifs within the differential regions increased by IFN treatment and/or USP18 KO. For scRNA-seq analysis, Data were mapped using Cell Ranger version 3.0.2 (10X Genomics) to mm10 and analyzed using the Seurat R for single cell genomics. For FACS analysis, data were analyzed using Flowjo version 10.

For manuscripts utilizing custom algorithms or software that are central to the research but not yet described in published literature, software must be made available to editors and reviewers. We strongly encourage code deposition in a community repository (e.g. GitHub). See the Nature Research guidelines for submitting code & software for further information.

Data

Policy information about availability of data

All manuscripts must include a data availability statement. This statement should provide the following information, where applicable:

- Accession codes, unique identifiers, or web links for publicly available datasets
- A list of figures that have associated raw data
- A description of any restrictions on data availability

The all data in this study are available.
High-throughput sequencing data generated in this study have been deposited in NCBI Gene Expression Omnibus (GEO).
The sequencing and processed data are available at GSE165424 (AE9a bulk RNA-seq), GSE196581 (AE9a bulk RNA-seq for paired ATAC-seq), GSE165425 (AE9a scRNA-seq; Usp18 one allele depletion), GSE196582 (AE9a scRNA-seq; Usp18 two allele depletion), GSE196580 (AE9a ATAC-seq for paired RNA-seq), GSE165426 (THP-1 ATAC-seq), GSE165427 (THP-1 ChIP-seq), GSE165428 (THP-1 RNA-seq), GSE165429 (MDA-MB-231 RNA-seq). Code/script for scRNA-seq analysis was deposited in github.com/kei16-cell/AE9a-scRNAseq.
GSE165424; [https://www.ncbi.nlm.nih.gov/geo/query/acc.cgi?acc=GSE165424]
GSE165425; [https://www.ncbi.nlm.nih.gov/geo/query/acc.cgi?acc=GSE165425]
GSE165426; [https://www.ncbi.nlm.nih.gov/geo/query/acc.cgi?acc=GSE165426]
GSE165427; [https://www.ncbi.nlm.nih.gov/geo/query/acc.cgi?acc=GSE165427]
GSE165428; [https://www.ncbi.nlm.nih.gov/geo/query/acc.cgi?acc=GSE165428]
GSE165429; [https://www.ncbi.nlm.nih.gov/geo/query/acc.cgi?acc=GSE165429]
GSE196580; [https://www.ncbi.nlm.nih.gov/geo/query/acc.cgi?acc=GSE196580]
GSE196581; [https://www.ncbi.nlm.nih.gov/geo/query/acc.cgi?acc=GSE196581]
GSE196582; [https://www.ncbi.nlm.nih.gov/geo/query/acc.cgi?acc=GSE196582]
For heterozygous Usp18 depletion; [https://github.com/kei16-cell/AE9a-scRNAseq-Usp18-heterozygous-deletion]
For homozygous Usp18 depletion; [https://github.com/kei16-cell/AE9a-scRNAseq-Usp18-homozygous-deletion

Field-specific reporting

Please select the one below that is the best fit for your research. If you are not sure, read the appropriate sections before making your selection.

☒ Life sciences ☐ Behavioural & social sciences ☐ Ecological, evolutionary & environmental sciences

For a reference copy of the document with all sections, see nature.com/documents/nr-reporting-summary-flat.pdf

Life sciences study design

All studies must disclose on these points even when the disclosure is negative.

Sample size	No sample size pre-calculation was performed as all sizes meet or exceed standards in this field. Mouse numbers for tumor study used were empirically derived from the literature published in murine cancer models such as Jiang, X., Hu, C., Arnovitz, S. et al. Nature communications 2016, doi: 10.1038/ncomms11452 for AML study, Song, W., Shen, L., Wang, Y. et al. Nature communications 2018, doi: 10.1038/s41467-018-04605-x, Rossi, A., Pakhomova, O.N., Pakhomov, A.G. et al. Scientific Reports 2019, doi: 10.1038/s41598-018-36527-5 for solid tumor study. We used at least 5 mice per group for tumor (AML and solid) study, and 5 mice per group were reproducible in follow up repeat experiments and in across tumor models. Sample size for in vitro experiments were conducted at n =/> 2 according to historical experience with data distribution in experiments such as qPCR. The sample sizes were made as large as possible and data was routinely collected across at least two independent experiments.
Data exclusions	No data was excluded with the exception of mouse studies. For all animal experiments, animals were only excluded if they died or had to be sacrificed according to the pre-defined criteria listed in the IACUC protocol.
Replication	All in vivo experiments were highly reproducible and were independently repeated at least 2 times. RNA-seq were performed using 3 biological replicates. ATAC-seq and ChIP-seq were performed using 2 biological replicates. All replication for RNA-seq, ATAC-seq and ChIP-seq were successful. All other in vitro experiments were performed at least twice independently and highly reproduced.
Randomization	For the AML mouse models, AML bearing mice were divided into groups have similar GFP(AML) distribution by checking GFP in peripheral blood before Usp18 deletion or Plk2 induction.
Blinding	Blinding was not performed in any experiments for AML mouse models because the data was generated by FACS (GFP) in blood and survival with or without Usp18 depletion by Oil or Tamoxifen injection. For the solid tumor mouse study, investigators were blinded to group allocation during data collection.

Reporting for specific materials, systems and methods

We require information from authors about some types of materials, experimental systems and methods used in many studies. Here, indicate whether each material, system or method listed is relevant to your study. If you are not sure if a list item applies to your research, read the appropriate section before selecting a response.

Materials & experimental systems

n/a	Involved in the study
☐	☒ Antibodies
☐	☒ Eukaryotic cell lines
☒	☐ Palaeontology and archaeology
☐	☒ Animals and other organisms
☒	☐ Human research participants
☒	☐ Clinical data
☒	☐ Dual use research of concern

Methods

n/a	Involved in the study
☐	☒ ChIP-seq
☐	☒ Flow cytometry
☒	☐ MRI-based neuroimaging

Antibodies

Antibodies used

The following antibodies for mouse experiments are from BioXCell.
anti-mouse-IgG1 (clone MOPC-21, Cat#BE0083, 0.25 mg/mouse)
anti-mouse-IFNAR1 (clone MAR1-5A3, Cat#BE0241, 0.25 mg/mouse)
The following antibodies for mouse experiments are from BioLegend.
CD3 (clone 17A2, Cat#100217, dilution 1:100)
CD3 (clone 17A2, Cat#100241, dilution 1:100)
CD4 (clone GK1.5, Cat#100433, dilution 1:100)
CD4 (clone RM4-5, Cat#100542, dilution 1:100)
CD8a (clone 53-6.7, Cat#100733, dilution 1:100)
CD8a (clone 53-6.7, Cat#100710, dilution 1:100)
CD11b (clone M1/70, Cat#101227, dilution 1:100)
CD11b (clone M1/70, Cat#101256, dilution 1:100)
CD11b (clone M1/70, Cat#101257, dilution 1:100)
CD11c (clone N418, Cat#117336, dilution 1:100)
Gr-1 (clone RB6-8C5, Cat#108427, dilution 1:100)
Gr-1 (clone RB6-8C5, Cat#108452, dilution 1:100)
B220 (clone RA3-6B2, Cat#103235, dilution 1:100)
CD19 (clone 6D5, Cat#115533, dilution 1:100)
CD19 (clone 6D5, Cat#115541, dilution 1:100)
Ter119 (clone TER-119, Cat#116227, dilution 1:100)
c-Kit (clone 2B8, Cat#105813, dilution 1:100)
Sca-1 (clone E13-161.7, Cat#122511, dilution 1:100)
CD150 (clone TC15-12F12.2, Cat#115907, dilution 1:100)
CD93 (clone AA4.1, Cat#136509, dilution 1:100)
CD16/32 (clone 93, Cat#101320, dilution 1:100)
CD45.2 (clone 104, Cat#109822, dilution 1:100)
CD25 (clone PC61, Cat#102008, dilution 1:100)
CD69 (clone H1.2F3, Cat#104541, dilution 1:100)
CD71 (clone RI7217, Cat#113813, dilution 1:100)
F4/80 (clone BM8, Cat#123137, dilution 1:100)
NK1.1 (clone PK136, Cat#108737, dilution 1:100)
Live/Dead (Cat#423106)
The following antibodies for mouse experiments are from eBioscience.
CD48 (clone HM48.1, Cat#12-0481-82, dilution 1:100)
CD8a (clone 4SM16, Cat#14-0195-82, dilution 1:100, for IHC)
Foxp3 (clone FJK-16s, Cat#17-5773-82, dilution 1:100)
The following antibodies for mouse experiments were from BD.
CD19 (clone 1D3, Cat#5537855, dilution 1:100)
The following antibodies were from Invitrogen.
streptavidin-APC-Alexa Fluor 750 (Cat#SA1027, dilution 1:100)
The following antibodies were from Cell signaling technology.
STAT1 (Cat#9172, dilution 1:1000) for both mouse and human.
p-STAT1 (Cat#9167, dilution 1:1000) for both mouse and human.
PLK2 (Cat#14812, dilution 1:1000) for human.
H2AX (Cat#2595, dilution 1:1000) for both mouse and human.
?H2AX (Cat#9718, dilution 1:1000) for both mouse and human.
LC3B (Cat#3868, dilution 1:1000) for human.
AIM2 (Cat#12948, dilution 1:1000) for human.
GSDMD (Cat#96458, dilution 1:1000) for both mouse and human.
GSDMD (Cat#39754, dilution 1:1000) for both mouse and human.
Caspase3 (Cat#9662, dilution 1:1000) for human.
Cleaved Caspase1 (Cat#4199, dilution 1:1000) for human.
NF-kB p65 (Cat#8242, dilution 1:1000) for human.
IRF9 (Cat#76684, dilution 1:1000) for human.
Calreticulin (Cat#19780, dilution 1:100) for both mouse and human.
HMGB1 (Cat#6893, dilution 1:100, for IHC) for mouse.
The following antibody was from GeneTex.
p84 (Cat#GTX70220, dilution 1:1000) for human.

GAPDH (Cat#GTX627408, dilution 1:1000) for human.
The following antibody was from Abcam.
GSDME (ab215191, dilution 1:1000) for both mouse and human.
The following antibodies were from Santacruz.
Caspase1 (sc-56036, dilution 1:1000) for human.
Myc (clone 9E10, sc-40, dilution 1:1000)
Snk (PLK2) (clone E-10, sc-374643, dilution 1:1000) for mouse.
The following antibody was from Sigma.
FLAG (F3165, M2, dilution 1:1000)
The following antibody was from Active Motif.
H3K27ac (Cat#91193, 4ug per ChIP) for human.
H3K27ac (Cat#39133, 4ug per ChIp) for mouse splenocytes
Anti-murine ISG15, human USP18, and murine USP18 were previously described and validated.

Validation

Specific validation information for all commercially available in this study can be found at their respective websites as below.
anti-mouse-IgG1
https://bioxcell.com/invivomab-mouse-igg1-isotype-control-unknown-specificity-be0083
anti-mouse-IFNAR1
https://bioxcell.com/invivomab-anti-mouse-ifnar-1-be0241
CD3
https://www.biolegend.com/en-us/products/percp-cyanine5-5-anti-mouse-cd3-antibody-5596?GroupID=GROUP20
CD3
https://www.biolegend.com/fr-fr/products/brilliant-violet-711-anti-mouse-cd3-antibody-10022
CD4
https://www.biolegend.com/en-us/products/percp-cyanine5-5-anti-mouse-cd4-antibody-4220
CD4
https://www.biolegend.com/fr-fr/products/brilliant-violet-570-anti-mouse-cd4-antibody-7379
CD8a
https://www.biolegend.com/en-us/products/percp-cyanine5-5-anti-mouse-cd8a-antibody-4255
CD8a
https://www.biolegend.com/fr-fr/products/pe-cyanine5-anti-mouse-cd8a-antibody-156
CD11b
https://www.biolegend.com/en-us/products/percp-cyanine5-5-anti-mouse-human-cd11b-antibody-4257
CD11b
https://www.biolegend.com/fr-fr/products/pe-dazzle-594-anti-mouse-human-cd11b-antibody-9826
CD11b
https://www.biolegend.com/fr-fr/products/brilliant-violet-605-anti-mouse-human-cd11b-antibody-7637
CD11c
https://www.biolegend.com/fr-fr/products/brilliant-violet-785-anti-mouse-cd11c-antibody-7963
Gr-1
https://www.biolegend.com/en-us/products/percp-cyanine5-5-anti-mouse-ly-6g-ly-6c-gr-1-antibody-4286?GroupID=GROUP20
Gr-1
https://www.biolegend.com/fr-fr/products/pe-dazzle-594-anti-mouse-ly-6g-ly-6c-gr-1-antibody-10319
B220
https://www.biolegend.com/en-us/products/percp-cyanine5-5-anti-mouse-human-cd45r-b220-antibody-4267
CD19
https://www.biolegend.com/en-us/products/percp-cyanine5-5-anti-mouse-cd19-antibody-4261?GroupID=GROUP20
CD19
https://www.biolegend.com/fr-fr/products/brilliant-violet-650-anti-mouse-cd19-antibody-7851
Ter119
https://www.biolegend.com/en-us/products/percp-cyanine5-5-anti-mouse-ter-119-erythroid-cells-antibody-4292
c-Kit
https://www.biolegend.com/en-us/products/pe-cyanine7-anti-mouse-cd117-c-kit-antibody-1900
Sca-1
https://www.biolegend.com/en-us/products/apc-anti-mouse-ly-6a-e-sca-1-antibody-3897?GroupID=BLG5162
CD150
https://www.biolegend.com/en-us/products/biotin-anti-mouse-cd150-slam-antibody-2619
CD93
https://www.biolegend.com/en-us/search-results/apc-anti-mouse-cd93-aa4-1-early-b-lineage-antibody-6621?GroupID=BLG8671
CD16/32
https://www.biolegend.com/fr-fr/products/trustain-fcx-anti-mouse-cd16-32-antibody-5683
CD45.2
https://www.biolegend.com/fr-fr/products/alexa-fluor-700-anti-mouse-cd45-2-antibody-3393
CD25
https://www.biolegend.com/fr-fr/products/pe-anti-mouse-cd25-antibody-424
CD69
https://www.biolegend.com/fr-fr/products/brilliant-violet-650-anti-mouse-cd69-antibody-13310
CD71
https://www.biolegend.com/fr-fr/products/brilliant-violet-421-anti-mouse-cd71-antibody-7304
F4/80
https://www.biolegend.com/fr-fr/products/brilliant-violet-421-anti-mouse-f4-80-antibody-7199
NK1.1
https://www.biolegend.com/fr-fr/products/brilliant-violet-510-anti-mouse-nk-1-1-antibody-8615
Live/Dead

https://www.biolegend.com/fr-fr/products/zombie-nir-fixable-viability-kit-8657
CD48
https://www.thermofisher.com/antibody/product/12-0481-82.html?ef_id=EAIaIQobChMIz8zD3Lqf9wIVKB-tBh3mDA8zEAAYASAAEgKF9PD_BwE:G:s&s_kwcid=AL!3652!3!278870232429!!!g!!
&cid=bid_pca_frg_r01_co_cp1359_pjt0000_bid00000_0se_gaw_dy_pur_con&gclid=EAIaIQobChMIz8zD3Lqf9wIVKB-tBh3mDA8zEAAYASAAEgKF9PD_BwE
CD8a for IHC
https://www.thermofisher.com/antibody/product/CD8a-Antibody-clone-4SM16-Monoclonal/14-0195-82
Foxp3
https://www.thermofisher.com/antibody/product/FOXP3-Antibody-clone-FJK-16s-Monoclonal/15-5773-82
CD19
https://www.bdbiosciences.com/en-nz/products/reagents/flow-cytometry-reagents/research-reagents/single-color-antibodies-ruo/fitc-rat-anti-mouse-cd19.553785
streptavidin-APC-Alexa Fluor 750
https://www.thermofisher.com/order/catalog/product/SA1027
STAT1
https://www.cellsignal.com/products/primary-antibodies/stat1-antibody/9172
p-STAT1
https://www.cellsignal.com/products/primary-antibodies/phospho-stat1-tyr701-58d6-rabbit-mab/9167
PLK2
https://www.cellsignal.com/products/primary-antibodies/plk2-d5r2b-rabbit-mab/14812
H2AX
https://www.cellsignal.com/products/primary-antibodies/histone-h2a-x-antibody/2595
[illegible]H2AX
https://www.cellsignal.com/products/primary-antibodies/phospho-histone-h2a-x-ser139-20e3-rabbit-mab/9718
LC3B
https://www.cellsignal.com/products/primary-antibodies/lc3b-d11-xp-rabbit-mab/3868
AIM2
https://www.cellsignal.com/products/primary-antibodies/aim2-d5x7k-rabbit-mab/12948
GSDMD
https://www.cellsignal.com/products/primary-antibodies/gasdermin-d-antibody/96458
GSDMD
https://www.cellsignal.com/products/primary-antibodies/gasdermin-d-e9s1x-rabbit-mab/39754
Caspase3
https://www.cellsignal.com/products/primary-antibodies/caspase-3-antibody/9662
Cleaved Caspase1
https://www.cellsignal.com/products/primary-antibodies/cleaved-caspase-1-asp297-d57a2-rabbit-mab/4199
NF-[illegible]B p65
https://www.cellsignal.com/products/primary-antibodies/nf-kb-p65-d14e12-xp-rabbit-mab/8242
IRF9
https://www.cellsignal.com/products/primary-antibodies/irf-9-d2t8m-rabbit-mab/76684
Calreticulin
https://www.cellsignal.com/products/antibody-conjugates/calreticulin-d3e6-xp-rabbit-mab-pe-conjugate/19780
HMGB1 for IHC
https://www.cellsignal.com/products/primary-antibodies/hmgb1-d3e5-rabbit-mab/6893
p84
https://www.genetex.com/Product/Detail/Nuclear-Matrix-Protein-p84-antibody-5E10/GTX70220
GAPDH
https://www.genetex.com/Product/Detail/GAPDH-antibody-GT239/GTX627408
GSDME
https://www.abcam.com/dfna5gsdme-antibody-epr19859-n-terminal-ab215191.html
Caspase1
https://www.scbt.com/ja/p/caspase-1-antibody-14f468
Myc
https://www.scbt.com/ja/p/c-myc-antibody-9e10
Snk (PLK2)
https://www.scbt.com/p/snk-antibody-e-10
FLAG
https://www.sigmaaldrich.com/catalog/product/sigma/f3165?lang=en&region=US
H3K27ac
https://www.activemotif.com/documents/tds/91193.pdf
H3K27ac
https://www.activemotif.com/catalog/details/39133

All antibodies were validated by the supplier and were checked in the lab by comparing to the manufacturer's or in-house results.
Statement from Bio X Cell: Our InVivoPlus™ antibodies feature all the great qualities of our InVivoMab™ antibodies. Statement from Bio X Cell: Our InVivoPlus™ antibodies feature all the great qualities of our InVivoMab™ antibodies. The InVivoPlus™ versions of our products are structurally and functionally identical to the InVivoMab™ versions with the added benefit of additional QC measures. InVivoPlus™ antibodies are screened for murine pathogens using ultrasensitive qPCR, screened for protein aggregation via dynamic light scattering, feature advanced binding validation via flow cytometry, ELISA, and/or Western blot, and are guaranteed to contain less than 1 endotoxin unit per milligram. Our InVivoPlus™ line of antibodies are designed to exceed the strict demands and rigorous standards required for in vivo work at any research organization.
Statement from BioLegend: BioLegend antibodies undergo an extensive series of testing to ensure quality at every step in the manufacturing process, as well as maintaining quality after the sale.
Statement from ThermoFisher Scientific (including Invitrogen/eBioscience/BD): Thermo Fisher Scientific is committed to adopting validation standards for our Invitrogen antibody portfolio. The Advanced Verification badge is applied to products that have passed

application and specificity testing. This badge can be found in the search results and at the top of the product specific webpages. Data supporting the Advanced Verification badges can be found in product specific data galleries.
Statement from Cell Signaling Technology: https://www.cellsignal.com/contents/our-approach-antibody-validation-principles/antibody-validation-for-immunofluorescence/ourapproach-validation-if
Statement from GeneTex: GeneTex understands the absolute necessity for reliable antibodies to achieve accurate and reproducible experimental results. To optimize the performance of our reagents, we employ various analytic validation strategies to ensure both consistent quality (see GeneTex's Approach to Antibody Lot-to-Lot Variability) and specificity. These modalities are in line with guidelines described by the International Working Group on Antibody Validation (IWGAV) and have become fundamental components of our quality assurance process: https://www.genetex.com/MarketingMaterial/Index/five_pillars
Statement from abcam: Antibody specificity is confirmed by looking at cells that either do or do not express the target protein within the same tissue. Initially, our scientists will review the available literature to determine the best cell lines and tissues to use for validation.
Validation for Santacruz antibodies for our study; These antibodies (anti-Myc, anti-caspase-1 and anti-snk) were cited in severl papers and we also validated in our hand.
Validation for Sigma; https://www.sigmaaldrich.com/US/en/technical-documents/technical-article/protein-biology/immunohistochemistry/antibody-enhanced-validation
Statement from Active Motif; Active Motif has high standards for the antibodies we manufacture. From target selection and immunogen design to the initial screening by dot blot and/or Western blot, we apply the same stringent guidelines that you would if you were making an antibody in your lab. Because ChIP is such a demanding technique, our ChIP validated antibodies have to meet an even higher standard. For our in house ChIP validated polyclonals, we inject many rabbits because we expect most of the antibodies produced will not meet our demanding criteria.
Finding the proper model system for ChIP validation can be tricky, requiring the right combination of cell type, gene target and even cell growth or stimulation. We have used both endpoint and real time quantitative PCR for ChIP validation, but have now gone exclusively to qPCR, as it allows us to measure the performance of specific antibody lots or formulations and compare them to each other for optimizationhttps://www.activemotif.com/chromatin-ip-antibody-validation

Eukaryotic cell lines

Policy information about cell lines

Cell line source(s)	293T, B16F10, THP-1, MV4-11, MOLM13, OCI-AML3, and Kasumi-1 were purchased from ATCC. MC38 was from National Cancer Institute (NCI). MDA-MB-231 which was originally from ATCC was kindly provided by Dr. David Cheresh. U5A (IFNAR2-/-) and U6A (STAT2-/-) cells were (kindly provided by Dr. George Stark.
Authentication	Authentication was not performed.
Mycoplasma contamination	293T, THP-1, U series cells, and MDA-MB-231 cells were validated mycoplasma-free by mycoplasma detection kit (Lonza). Others were not tested.
Commonly misidentified lines (See ICLAC register)	No ICLAC cell lines were used

Animals and other organisms

Policy information about studies involving animals; ARRIVE guidelines recommended for reporting animal research

Laboratory animals	Conditional Usp18 knockout mice (Usp18f/f) were generated in this study and crossed with UBCER-Cre mice (Stock 008085, B6.Cg-Tg-Ndor1(UBC-cre/ERT2)1Ejb/2J. The Jackson Laboratory). C57BL/6 background Usp18+/- mice were previously described. B6.SJL-Ptprca Pepcb/BoyJ (CD45.1) (Stock 002014) mice from The Jackson Laboratory were used as recipients for the competitive transplantation experiment. C57BL/6 WT mice were used as recipients for all transplantation experiments. All mice in this study are both sex 8-12 weeks age were used. Animal vivarium condition; light/dark cycle: 12 hours, temperature: 18-23 degrees C, humidity: 40-60 %.
Wild animals	This study did not involve wild animals.
Field-collected samples	This study did not involve field collected samples.
Ethics oversight	All the animal studies were approved by the University of California San Diego (UCSD) Institutional Animal Care and Use Committee (IACUC, protocol # S07271); all experiments in this study adhere with all relevant ethical regulations for animal research.

Note that full information on the approval of the study protocol must also be provided in the manuscript.

ChIP-seq

Data deposition

☒ Confirm that both raw and final processed data have been deposited in a public database such as GEO.

☒ Confirm that you have deposited or provided access to graph files (e.g. BED files) for the called peaks.

Data access links *May remain private before publication.*	GEO accession number is GSE165427; [https://www.ncbi.nlm.nih.gov/geo/query/acc.cgi?acc=GSE165427]

Files in database submission	GSM5033140 THP-1_WT0hIP_rep1_ChIP-seq<br>GSM5033141 THP-1_WT0hIP_rep2_ChIP-seq<br>GSM5033142 THP-1_WT0hinp_rep1_ChIP-seq<br>GSM5033143 THP-1_WT0hinp_rep2_ChIP-seq<br>GSM5033144 THP-1_WT6hIP_rep1_ChIP-seq<br>GSM5033145 THP-1_WT6hIP_rep2_ChIP-seq<br>GSM5033146 THP-1_WT6hinp_rep1_ChIP-seq<br>GSM5033147 THP-1_WT6hinp_rep2_ChIP-seq<br>GSM5033148 THP-1_KO0hIP_rep1_ChIP-seq<br>GSM5033149 THP-1_KO0hIP_rep2_ChIP-seq<br>GSM5033150 THP-1_KO0hinp_rep1_ChIP-seq<br>GSM5033151 THP-1_KO0hinp_rep2_ChIP-seq<br>GSM5033152 THP-1_KO6hIP_rep1_ChIP-seq<br>GSM5033153 THP-1_KO6hIP_rep2_ChIP-seq<br>GSM5033154 THP-1_KO6hinp_rep1_ChIP-seq<br>GSM5033155 THP-1_KO6hinp_rep2_ChIP-seq
Genome browser session (e.g. UCSC)	Genome browser session for ChIP-seq in this study is at https://genome.ucsc.edu/s/ttroutman/KeiichiroArimoto

Methodology

Replicates	2 biological replicates.
Sequencing depth	Sequencing condition; Paired-end 150bp<br>GSM5033140 THP-1_WT0hIP_rep1_ChIP-seq total (27055205), mapped(23039236)<br>GSM5033141 THP-1_WT0hIP_rep2_ChIP-seq total (31096230), mapped(26264089)<br>GSM5033142 THP-1_WT0hinp_rep1_ChIP-seq total (32991618), mapped(26716001)<br>GSM5033143 THP-1_WT0hinp_rep2_ChIP-seq total (29102403), mapped(23590703)<br>GSM5033144 THP-1_WT6hIP_rep1_ChIP-seq total (28154521), mapped(23990697)<br>GSM5033145 THP-1_WT6hIP_rep2_ChIP-seq total (27870579), mapped(23233309)<br>GSM5033146 THP-1_WT6hinp_rep1_ChIP-seq total (33155428), mapped(26843124)<br>GSM5033147 THP-1_WT6hinp_rep2_ChIP-seq total (30670104), mapped(24641137)<br>GSM5033148 THP-1_KO0hIP_rep1_ChIP-seq total (33572296), mapped(27234108)<br>GSM5033149 THP-1_KO0hIP_rep2_ChIP-seq total (31910030), mapped(25845317)<br>GSM5033150 THP-1_KO0hinp_rep1_ChIP-seq total (25601739), mapped(20316636)<br>GSM5033151 THP-1_KO0hinp_rep2_ChIP-seq total (29479029), mapped(23407414)<br>GSM5033152 THP-1_KO6hIP_rep1_ChIP-seq total (34451013), mapped(27868556)<br>GSM5033153 THP-1_KO6hIP_rep2_ChIP-seq total (34932516), mapped(27764395)<br>GSM5033154 THP-1_KO6hinp_rep1_ChIP-seq total (33407905), mapped(26607924)<br>GSM5033155 THP-1_KO6hinp_rep2_ChIP-seq total (31879434), mapped(25222109)
Antibodies	H3K27ac antibody (Active Motif #91193)
Peak calling parameters	The FASTQ files from ChIP-seq experiments were mapped to the human hg38 genome using Bowtie2 with default parameters. HOMER software was used for further analysis. First, pooled ATAC-seq peaks were sorted into promoter proximal or promoter distal peaks using a cutoff of within 3000 bp of the transcription start site. Then H3K27ac ChIP-seq data was quantified at each peak using annotatePeaks.pl within a 1000 bp window of the ATAC-seq peak, as described previously (PMCID: PMC7305990). Differentially acetylated regions were identified using DeSeq2 through HOMER getDiffExpression.pl default parameters (fold change > 2 and adjusted p-value < 0.05). Next, peak windows were shifted back to the original size of the ATAC-seq data and findMotifsGenome.pl was used to assess enrichment of known DNA motifs within the differential regions increased by IFN treatment and/or USP18 KO.
Data quality	HOMER performs multiple filtering for peak identification and FDR for peak identification is 0.1 %.
Software	Bowtie2 HOMER

Flow Cytometry

Plots

Confirm that:

☒ The axis labels state the marker and fluorochrome used (e.g. CD4-FITC).

☒ The axis scales are clearly visible. Include numbers along axes only for bottom left plot of group (a 'group' is an analysis of identical markers).

☒ All plots are contour plots with outliers or pseudocolor plots.

☒ A numerical value for number of cells or percentage (with statistics) is provided.

Methodology

Sample preparation	The blood was lysed using RBC lysis buffer (made in house) and the remaining cells were analyzed.

Sample preparation	Spleens were harvested from mice and collected in FACS buffer. Single cells suspension was obtained by pushing the organ through a 40 um sieve with a syringe plunger. Single cells suspension from B16F10 tumor was obtained by pushing the tumor through a 40 um sieve with a syringe plunger. For the MC38 tumor, we used collagenase I for 1 hour at 37 degree before pushing the tumor through a 40 um sieve with a syringe plunger. The blood was removed with RBC lysis buffer (made in house) and the cells were stained with fluorescent Ab for FACS analysis.<br>The blood was collected from mice with mandible bleeds following guidelines and procedure from the Institute Animal Ethics Committee.
Instrument	BD FACSCanto , BD FACSAria-II, NovoCyte Advanteon
Software	BD FACSDIVA, BD Flowjo
Cell population abundance	The sort purity was > 95%. The percentage of each population sorted is shown in the relevant section of the paper.
Gating strategy	The cells were firstly gated on a size using SSC-A/FSC-A, then on a single cell gate based on FSC-W/FSC-H, SSC-W/SSC-H, and then on a live cell gate based on PI negative cells. Cell death was analyzed by Annexin V-APC and PI. Examples for all the gates subsequent to live cells are provided in Supplementary Figure 12.

☒ Tick this box to confirm that a figure exemplifying the gating strategy is provided in the Supplementary Information.
